# Implementation conditions for diet and physical activity interventions and policies: an umbrella review

**DOI:** 10.1186/s12889-015-2585-5

**Published:** 2015-12-17

**Authors:** Karolina Horodyska, Aleksandra Luszczynska, Catherine B. Hayes, Miriam P. O’Shea, Lars J. Langøien, Gun Roos, Matthijs van den Berg, Marieke Hendriksen, Ilse De Bourdeaudhuij, Johannes Brug

**Affiliations:** Department of Psychology, SWPS University of Social Sciences and Humanities, 30b Ostrowskiego St, 53238 Wroclaw, Poland; Trauma, Health, & Hazards Center, University of Colorado, 1861 Austin Bluffs Pkwy, Colorado Springs, CO 80933-7150 USA; Department of Public Health and Primary Care, Trinity College Dublin, Centre for Health Sciences, Tallaght Hospital, Dublin 24, Ireland; Department for Physical Education, Norwegian School of Sport Sciences, P.O. BOX 4014, Ullevål Stadion, N-0806 Oslo, Norway; SIFO – National Institute for Consumer Research, P.O. BOX 4682, Nydalen, N-0405 Oslo, Norway; National Institute for Public Health and the Environment, Antonie van Leeuwenhoeklaan 9, 3721 Bilthoven, The Netherlands; Department of Movement and Sport Sciences, Ghent University, Watersportlaan 2, 9000 Ghent, Belgium; VU University Medical Center, Amsterdam, Van der Boechorststraat 7, 1081 BT Amsterdam, The Netherlands

**Keywords:** Implementation, Systematic review, Policy, Intervention, Diet, Physical activity, Sedentary behavior, RE-AIM

## Abstract

**Background:**

This umbrella review aimed at identifying evidence-based conditions important for successful implementation of interventions and policies promoting a healthy diet, physical activity (PA), and a reduction in sedentary behaviors (SB). In particular, we examined if the implementation conditions identified were intervention-specific or policy-specific. This study was undertaken as part of the DEterminants of DIet and Physical Activity (DEDIPAC) Knowledge Hub, a joint action as part of the European Joint Programming Initiative a Healthy Diet for a Healthy Life.

**Methods:**

A systematic review of reviews and stakeholder documents was conducted. Data from nine scientific literature databases were analyzed (95 documents met the inclusion criteria). Additionally, published documentation of eight major stakeholders (e.g., World Health Organization) were systematically searched (17 documents met the inclusion criteria). The RE-AIM framework was used to categorize elicited conditions. Across the implementation conditions 25 % were identified in at least four documents and were subsequently classified as having obtained sufficient support.

**Results:**

We identified 312 potential conditions relevant for successful implementation; 83 of these received sufficient support. Using the RE-AIM framework eight implementation conditions that obtained support referred to the reach in the target population; five addressed efficacy of implementation processes; 24 concerned adoption by the target staff, setting, or institutions; 43 referred to consistency, costs, and adaptations made in the implementation process; three addressed maintenance of effects over time. The vast majority of implementation conditions (87.9 %; 73 of 83) were supported by documents referring to both interventions and policies. There were seven policy-specific implementation conditions, which focused on increasing complexities of coexisting policies/legal instruments and their consequences for implementation, as well as politicians’ collaboration in implementation.

**Conclusions:**

The use of the proposed list of 83 conditions for successful implementation may enhance the implementation of interventions and policies which pursue identification of the most successful actions aimed at improving diet, PA and reducing SB.

**Electronic supplementary material:**

The online version of this article (doi:10.1186/s12889-015-2585-5) contains supplementary material, which is available to authorized users.

## Background

Positive changes in diet, physical activity (PA), and sedentary behaviors (SB) may serve the same health-related goals such as maintaining healthy body weight, reducing risk for non-communicable diseases (including cancer, type 2 diabetes, cardiovascular diseases) and improving health in general [1]. These behaviors therefore may be jointly addressed in guidelines issued by major health organizations and they may be targeted jointly in interventions and policies [1, 2]. Health promotion efforts are directed at developing interventions and policies that result in significant and sustainable changes in dietary, physical activity (PA), and sedentary behaviors (SB) [3]. Policies and interventions are formed as purposive courses of actions to promote such positive changes in these behaviors [4]. Both policies and interventions may target individuals’ skills or beliefs, address contexts such as social systems, the physical or built environment, or create opportunities for practicing the behavior [5]. Conceptual and definitional distinctions between policies and interventions may be arbitrary. While both are seen as approaches to support initiation and maintenance of health-promoting behavior, sometimes in practice it might be considered that a specific intervention, if implemented at national level, would become recognized as a policy whereas some policies implemented locally would become recognized as interventions. Although several health promotion programs may be difficult to classify unequivocally as either polices or interventions, major health organizations accentuate the fact that policies and interventions may require different good practice guidelines [4]. For the purpose of this study policies are defined as actions formulated in a specific political process, adopted, and enforced by regional, national, or international public agencies, whereas interventions are defined as actions not yet endorsed, enabled or executed by regional, national, or international agencies [4].

In order to improve evidence-based health promotion, researchers and practitioners need to know *if* the intervention/policy works [6] and if so *how* it works. The *how* aspect may refer to the behavior change techniques, active components of interventions/policies, and underlying processes which explain behavior change [7, 8]. Additionally, the ‘*how*’ aspect may refer to the main operational characteristics of intervention and policies, such as content development (and its management), the use of theory, deciding the target group, target behavior, setting, and practitioners [2, 9]. The *if* aspect of interventions and policies promoting healthy diet and PA may encompass the facets of monitoring and evaluation, such as selection of outcomes, evaluation of effects, time when effects are observed, and effect size [2, 9]. Those aspects are covered in frameworks guiding the development of behavior change interventions and policies, such as the Behavior Change Wheel [10] and in the reporting guidelines for behavior change interventions and policies, such as WIDER [7].

A third focal point in the process of the development and evaluation of successful interventions and policies concerns *the conditions for implementation* [2]. Interventions and policies that have been found to be efficacious will only make a true difference if these are implemented in the best possible way, that is with attention to implementation theories or frameworks, evidence-based best practice guidelines. The identification of the critical conditions for implementation, in particular the optimal ways to translate laboratory-based research into real-word settings*,* is key to developing successful interventions and policies [3]. Leading approaches fostering implementation, such as the Consolidated Framework for Implementation Research [11], capture the characteristics of implementation in addition to the *how* and *why* aspects (i.e., characteristics of the participants, setting, the content, the effectiveness, and the underlying mechanisms). Therefore, a definition of implementation conditions may be broad and encompass any characteristics which may have even a distant relationship to implementation actions. Our paper however, will use a narrower definition of implementation, proposed by World Health Organization [2]. According to this definition implementation conditions refer to the performance of implementation, program management, and participation processes [2].

There are at least 50 theoretical approaches and frameworks, which explain or enhance implementation of health promotion actions [12]. Tabak and her coauthors (2012) analyzed theories and frameworks in terms of their focus on implementation (in contrast to a focus on dissemination), flexibility of the constructs included in theories or frameworks (broad versus specific/operational), and socio-ecological feasibility (e.g., a potential to consider individual, organizational, and community factors). Among frameworks and theories which address implementation (in contrast to dissemination), and which provide a relatively detailed, step-by-step description of implementation conditions, only three tackle broad socio-ecological levels (i.e., address community, organizational, and individual factors). These are: the Ottawa Model for Research Use [13], the Precede-Proceed Model [14], and the RE-AIM framework [15]. The Ottawa and Precede-Proceed models [13, 14] offer a guide to a process of implementation and formulate a number of consecutive steps. These models are operational in character, as they specify the sequence of actions securing optimal implementation. In contrast, the RE-AIM framework [15, 16] defines five broad domains of implementation conditions. The RE-AIM was designed to enhance the quality and maximize the impact of actions translating research into practice. RE-AIM comprises five domains (1) Reach in the target population, (2) Efficacy, (3) Adoption by the target staff, setting, or institutions, (4) consistency, costs and adaptations made in the Implementation process, and (5) Maintenance of the effects in individuals and settings over time [15]. As the present study aims at eliciting and describing evidence-based implementation conditions, the RE-AIM framework [15] was identified as the most pragmatic due to its descriptive and categorical approach.

Several frameworks and theories only address implementation conditions for policies cf. [12, 17]. Developing separate conceptualizations for policies is guided by an assumption that implementation of policies may have distinct characteristics, compared to implementation of interventions. Some frameworks suggest that the difference may result from the salient role of the political context and a broader social context (encompassing for example economic factors and health services) which inform implementation of policies [17]. Identifying implementation conditions specific for policies may allow for an insight into distinct processes explaining successful policies, compared to processes responsible for a success of interventions. Importantly, the specificity assumptions made by the policy implementation frameworks have not been tested using methods of systematic reviews. To fill this gap, the present study investigates the actual differences in the empirical evidence for the role of implementation conditions for policies and for interventions (promoting healthy diet and physically active lifestyle and reducing sedentary behavior).

The number of research papers on implementation conditions is growing rapidly, with dozens of systematic reviews, position papers, and evidence-based stakeholders’ documents issued every year. These documents often rely on similar search or synthesis strategies but reach different conclusions. As yet, there is no overarching synthesis of the empirical evidence for implementation conditions in interventions and policies addressing dietary behavior, PA and SB.

### Aims

As the part of the investigation undertaken by the DEDIPAC project (the DEterminants of DIet and Physical Activity Knowledge Hub, the first Research Action of the European Union’s Joint Programming Initiative on Healthy Diet for Healthy Life) [18], the present study aimed to (1) identify conditions for successful implementation of interventions promoting healthy diet, PA and a reduction of SB and (2) to identify conditions for successful implementation of policies promoting healthy diet, PA, and a reduction of SB.

We investigated the implementation conditions in policies and interventions targeting the general population, children, adults, older adults and vulnerable populations. Applying the RE-AIM framework [15], we sought for evidence-based conditions of implementation which may refer to the domains of (1) reach, (2) efficacy (3) adoption (4) consistency, cost and adaptations in implementation, and (5) maintenance.

## Methods

### Materials and general procedures

To achieve the aims, we performed an umbrella review - i.e. a review of review documents - integrating evidence obtained from existing systematic reviews, position review papers, and stakeholders’ documents. Umbrella reviews represent a way of synthesizing the evidence accumulated in systematic reviews cf. [19]. A majority of umbrella reviews focus on analyzing materials obtained from systematic reviews cf. [20] however, the aim of this study required integrating the evidence presented in reviews with practice recommendations issued by major stakeholders cf. [9]. The questions and methods of this umbrella review were developed and approved using the rapid review approach [21]. Adherence to PRISMA guidelines [22] and the respective checklist is reported in Additional file 1. The study and its protocol were not registered. Protocols are available from the first and second author upon request.

Three types of documents were retrieved and analyzed in order to elicit the implementation conditions: (1) systematic reviews analyzing original research on implementation conditions for policies/interventions, (2) position papers that offered a comprehensive (but not systematic) review of research evidence on implementation conditions, and (3) documents issued by major national and international stakeholders providing evidence-based recommendations referring to implementation conditions. We investigated documents aimed at eliciting empirical evidence and evidence-based recommendations for policies and interventions targeting healthy diet, PA, or SB.

### Peer-reviewed documents: search strategy, inclusion, and exclusion criteria

The search was conducted in Medline, Embase, Cochrane Database of Systematic Reviews, PsycINFO, PsychArticles, Health Source: Nursing/Academic Edition, Academic Premier, Social Citation Index, and Scopus. Documents published between the inception of databases and August 2014 were included. Combinations of 4 groups of keywords were applied, referring to: (1) implementation conditions (implement* or disseminat* or translat* or “factors for” or transfer* or “external validity” or “recommendation* for practice”), (2) the type of action (intervention or polic* or “prevention program*”), (3) the design (“systematic review” or review or “meta-analys*”), and (4) the outcomes (“physical activity” or active or exercise or sedentary or diet or nutrition or fat or snack or fruit or vegetable or fiber or fibre or soda or meal or food or “energy intake” or calorie* or obes*). Two research teams conducted separate searches for (1) policy conditions [LJL and GR] and (2) intervention conditions [KH and AL]. At least two researchers were involved at all stages of data selection, data evaluation, and coding.

**Fig. 1:**
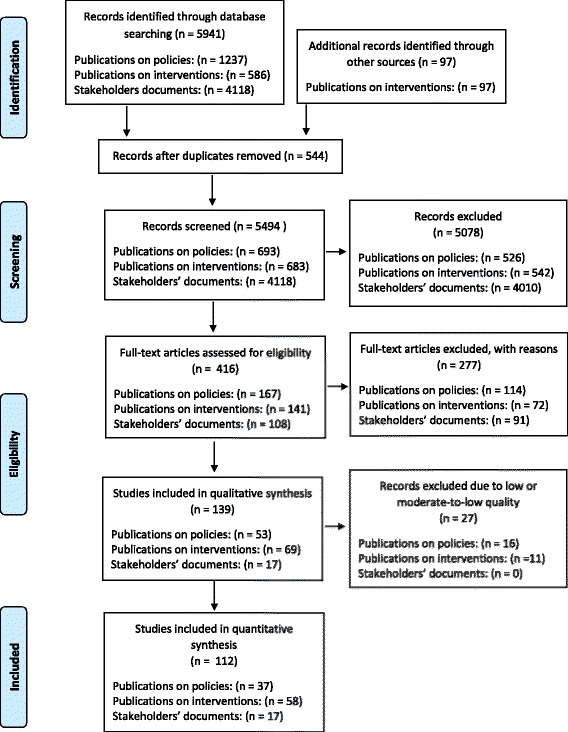
The flow chart. The selection process for peer-reviewed documents (policy documents, intervention documents, and stakeholders documents)

The stages of the data selection process are presented in Fig. 1, following the PRISMA template for reporting the results of systematic reviews [22]. The preliminary search yielded 1237 entries for policies and 586 entries for interventions (including papers which addressed both policies and interventions), which accounted for a combination of keywords in either title, abstract or keywords. Identified abstracts were then screened by two researchers (LJL and GR or KH and AL). We used manual searches of the reference lists to identify additional documents (*n* = 97).

The following documents were excluded: (a) dissertations, protocols, conference materials, and book chapters; (b) reviews which indicated a need for testing implementation conditions, but did not investigate implementation in the results sections; (c) publications addressing multi-behavior policies or interventions, which did not allow to identify whether specific implementation conditions were observed in policies/interventions aiming at either dietary behaviors or PA or SB; (d) documents that reviewed guidelines for diet, PA, or SB, but did not indicate implementation conditions; (e) publications which discussed only one example of policy or intervention; (f) reviews which analyzed theoretical approaches or frameworks rather than empirical evidence; (g) and reviews which analyzed qualitative studies only.

We included the documents reviewing empirical evidence for policies and interventions targeting healthy diet, PA, or SB. Only documents published in peer-reviewed English-language journals were included. In the case of *systematic reviews* we included reviews of quantitative studies using the following criteria for systematic reviews: clearly defined study aims, search strategy, inclusion and exclusion criteria, design of original studies, and a suitable synthesis given the heterogeneity of findings, cf. [23]. In case of *non-systematic position reviews*, papers focusing on reviewing evidence-based conditions for implementation were included. If several papers were published by the same authors on the same original studies, we included the most recent document, and sought for other (distinct) implementation conditions in earlier documents.

To account for the risk of bias in individual documents, *quality assessment* of each systematic review was conducted using the Methodological Quality Checklist (MQC) [24]. This is a 7-item scale with total scores ranging from 0 to 7. MQC evaluates strategies applied in original reviews and allocates a score of 1 to each of the following seven items: (1) well-defined study participants, intervention/policy, and outcomes; (2) several databases are searched; strategies for reference checking are used; (3) transparent inclusion and exclusion criteria; (4) the number and designs of original studies are clarified; (5) a quality assessment of original studies is included; (6) methods of data synthesis are specified and data heterogeneity is accounted for; and (7) at least two researchers are involved at each stage of review process. Previous umbrella reviews using MQC applied the cutoff of 4 as representing moderate or high quality [9, 20] and used the cutoff of 4 in MQC as the inclusion criterion threshold. Therefore only systematic reviews scoring ≥ 4, were included into the final analyses.

Additionally, to account for the risk of bias in individual documents prepared by stakeholders, the Methodological Quality Checklist for Stakeholder Documents and Position Papers, (MQC-SP; [9]) was used to evaluate the quality of peer-reviewed position papers. This scale tackles six quality criteria (major stakeholder involved, well-defined aim, robust methodology, quality evaluation of analyzed material, appropriate synthesis of analyzed material, more than one stakeholder or coauthors involved). The total scores range from 0 to 6. Only papers scoring ≥ 3, representing moderate or high quality, were included in the analyses.

Two researchers (LJL and GR or KH and AL) independently rated the quality of all documents. For both types of analyzed documents (systematic reviews, evidence-based position papers) the concordance of quality evaluations was high, with kappa (κ) coefficients of .91 to 1.00 (*p* < .001).

### Stakeholders’ documents (other than peer-reviewed): search strategy, inclusion criteria, exclusion criteria, and quality evaluation

To obtain *major stakeholders’ documents* aiming at eliciting implementation conditions, a group of eight experts used the consensus method [23]. The criteria for selecting the stakeholder documents were: (1) documents issued in the English language and available for download; (2) documents that provided evidence-based good practice recommendations for policies and interventions which addressed diet, PA or SB targeting any population, as the main outcome of the interventions/policies. Similar inclusion criteria were used in previous reviews of stakeholder documents [9, 25]. Publicly available document databases of the following stakeholders were reviewed: European Commission (EC), National Institute for Health and Clinical Excellence (NICE; United Kingdom), World Health Organization (WHO Global), Regional Office for Europe (WHO European Region), Centers for Disease Control and Prevention (CDC; USA), Institute of Medicine (IOM; USA), Australian Department of Health (ADH), and National Health and Medical Research Council (NHMRC; Australia).

A similar search strategy to that used for the database search was attempted in all of the stakeholder websites. However, due to the limited capacity of the search engines within the stakeholder websites a generic search engine (Google) was used in an attempt to elicit additional potentially relevant documents. All websites of respective stakeholders were searched using the same keywords as in the peer-reviewed documents reviews described above, accompanied by the names of the stakeholder organizations (or their acronyms). The initial search resulted in 4118 potentially relevant documents (Fig. 1**)**.

The potentially relevant documents were then screened by two researchers (MOS and CBH). We included documents which aimed at (1) reviewing conditions relating to implementation or (2) providing an overview of implementation conditions, or (3) formulating implementation recommendations in interventions or policies promoting healthy diet, PA, or a reduction of SB. Only documents developed and officially endorsed by a respective stakeholder were included. Documents were excluded if they presented preselected examples of implementation conditions (e.g., one condition only), instead of providing an overview of such conditions. Documents aiming at interventions or policies focusing on other main outcomes than healthy diet, PA or a reduction of SB (e.g. cardiovascular and cerebrovascular disease) were also excluded.

Finally, the quality of the documents was evaluated, using MQC-SP [9]. Two researchers (MOS and CBH) independently rated all stakeholders’ documents. Papers scoring ≥ 3, i.e. moderate to high quality, were included into the further analyses. The concordance of the quality evaluation was very high, with κ = 1.00, *p* < .001.

Overall, 17 stakeholder documents met all inclusion criteria and were further analyzed.

### Data extraction, coding, and synthesis

To ensure accuracy and consistency of data extraction and coding at least two researchers extracted and coded data independently. Any disagreements in the processes of data selection and abstraction were resolved by the consensus method (searching for possible rating errors, followed by a discussion and arbitration by a third researcher) [23].

*Descriptive data* (see Additional file 1) was extracted by one or two researchers (KH, LJL, or MOS) and then verified by another researcher (AL, GR, CBH or KH). Extracted data included: (1) the descriptive characteristics of the original studies (e.g., participants, target behavior) synthesized in the analyzed documents; (2) data necessary to evaluate the quality of the studies.

The *potential implementation conditions for interventions and policies* were extracted from each document. In particular, we retrieved the names of implementation conditions (as documented by authors of original documents) and their operationalization or definition. In the case of systematic reviews, only implementation conditions which were incorporated in the original analysis and supported by empirical evidence obtained in original trials were included. In the case of position papers, only conditions which were illustrated with empirical evidence were extracted. For both systematic reviews and position papers, if the implementation conditions were mentioned solely among the guidelines for future research or supported by theoretical frameworks only, they were not included. In case of stakeholders’ documents we retrieved implementation conditions which were operationalized and indicated as crucial for the implementation process.

As the focus of the present study was to analyze implementation conditions as narrowly defined by WHO [2], we excluded the conditions which addressed the main characteristics of interventions and policies (e.g., the content of the intervention/policy, the theory used in its development, characteristics of participants and target behaviors, general characteristics of practitioner and setting; cf. [9]. We also excluded characteristics referring to evaluation/monitoring of the effects of the interventions/policies (e.g., costs and funding for intervention/policy development, outcomes’ selection, evaluation of the influence on behavior, effect size, the evaluation of generalizability of the effects, active components and underlying processes; cf. [9]. Any of these characteristics may have an indirect effect on implementation processes or may be related to implementation conditions (e.g., an intervention which has only short-term effects on behavior may be related to participants’ evaluation of the intervention as less feasible; the costs of developing the policy may affects costs of its implementation). In contrast, we sought characteristics of interventions and policies which constitute the core conditions for implementation (e.g., the evaluation of the intervention as feasible; the cost of the process of implementation of the intervention/policy).

The implementation conditions that had an equivalent operationalization but different original names were considered to represent the same construct (e.g., attrition across the program conditions; attrition across the sub-types of the program). The findings are presented using definitions as presented by the authors of the original documents (see Additional file 1). Interventions and policies aimed at any type of PA (general levels of PA or its specific types, such as climbing stairs) were coded as referring to PA. Only five documents addressed SB. As it was considered that conditions for implementation of interventions/policies to address PA and SB may be comparable, PA and SB were combined into a single category. Interventions/policies targeting narrowly defined dietary behaviors (e.g., a change in fat intake) as well as addressing more complex dietary changes (e.g., total calorie intake) were coded as referring to dietary behavior.

In next step extracted implementation conditions were *allocated into the five domains* proposed in RE-AIM [15]. They were considered as representing (1) Reach, (2) Efficacy (3) Adoption (4) consistency, cost and adaptations in Implementation, or (5) Maintenance. For example 29 potential implementation conditions were identified for the Reach domain. The allocation was conducted by two researchers (KH and AL, or LJL and GR, or MOS and CBH).

Afterwards, conditions within each RE-AIM domain were combined into *broader thematic categories*. Implementation conditions allocated to these broader thematic categories shared at least one crucial aspect of implementation processes. For example all conditions related to recruitment process were combined into one category - ‘Strategies facilitating recruitment processes’; all conditions related to participation processes were grouped into one broader category - ‘Issues in participation processes and their effects on implementation’. Two researchers (KH, AL) independently clustered all identified implementation conditions into these broader categories identified within each RE-AIM domain. The names of broader categories and their contents were then independently evaluated by six researchers (MOS, CBH, LJL, GR, MH, and MvdB) who searched for flaws in categorization and evaluated the meaningfulness of broader categories.

Finally, the implementation condition was *categorized as an implementation condition which received sufficient support* if the respective condition was indicated in *at least four documents* (including systematic reviews, position review papers or stakeholders’ documents). Similar principal summary measures were used in previous umbrella reviews [9]. This threshold is based on the number of documents supporting each implementation condition and it represents the top quartile in the number of the supporting documents. Across implementation conditions, 75 % were supported by 1–3 documents, whereas 25 % were supported by at least four documents (Additional file 1). This arbitrary inclusion threshold was obtained in a consensus meeting, represented by the research groups from four countries involved in the DEDIPAC project. The impact on implementation conditions of increasing and decreasing this threshold was discussed. Similar summary measures, based on the upper quartile-based thresholds are used in health promotion research eliciting good practice characteristics [9, 25].

## Results

### Description of analyzed material

A total of 112 documents were included. The final selection consisted of 50 (44.6 %) systematic reviews, 17 (15.2 %) stakeholders’ documents, and 45 (40.2 %) position review papers (Additional file 1).

Systematic reviews investigated a total of 2094 original studies (see Additional file 1). The documents provided recommendations which could be applied to both policies and interventions (*k* = 37, 33 %), addressed interventions only (*k* = 43, 38.4 %), or focused on policies only (*k* = 32, 28.6 %). Regarding behaviors, 54 documents (48.2 %) referred to both PA and dietary behaviors, whereas 38 (33.9 %) focused on PA/SB only, and 20 (17.9 %) analyzed dietary behaviors only. Populations analyzed in original papers included: general population samples (*k* = 43, 38.3 %), children (*k* = 21, 18.8 %), children and adolescents (*k* = 15, 13.4 %), vulnerable populations, such as ethnic minorities or groups with low socio-economic status (*k* = 8, 7.1 %), adults with a chronic disease, including cardiovascular disease, diabetes, and cancer (*k* = 7, 6.3 %), adults (*k* = 6, 5.4 %), older adults (*k* = 7, 6.3 %), and adults at a workplace (*k* = 5, 4.4 %).

Based on the inclusion criteria, the quality of papers included in the analysis ranged from moderate to good (see Additional file 1). For systematic reviews, MQC scores ranged from 4 to 7 (*M* = 5.24, *SD* = 1.06). For position paper reviews and stakeholders’ documents MQC-SP scores ranged from 3 to 6 (*M* = 4.15, *SD* = 1.02).

### Implementation conditions

Overall, we identified 312 potential implementation conditions (see Additional file 1). The implementation conditions were supported by between 1 and 37 documents (*M* = 3.04, *SD* = 3.63. Among these, 83 (26.6 % of 312) implementation conditions received sufficient support (i.e., were indicated by at least four analyzed documents). The 229 remaining characteristics (see Additional file 1) fell below the threshold. Therefore they were not included into the final list of implementation conditions.

Table 1 yields the evidence supporting these 83 implementation conditions. Across the implementation conditions which received sufficient support, the vast majority (*n* = 73, 87.9 %) were generic i.e. the evidence for them was found in documents addressing interventions and policies. Only seven (8.4 %) implementation conditions were specific for policies and only three (3.6 %) were specific for interventions only.

**Table 1:** Implementation conditions for policies and interventions aiming at dietary behavior, physical activity, and sedentary behavior change: a synthesis of evidence

RE-AIM domain	Systematic reviews, stakeholders’ documents, and position reviews endorsing respective characteristics		
*Characteristics category*			
Implementation characteristics	Policies only	Interventions only	Policies and interventions
Domain: Reach			
(a) Strategies facilitating recruitment processes			
Resources/strategies for implementers helping them to invite and follow-up participants		Systematic reviews [5].	Systematic reviews [26, 27]; Position reviews [28].
Awareness raising (strategies to raise awareness of dietary behavior, physical activity, sedentary behaviors, as well as interventions and policies) to help implementers to invite participants	Systematic reviews [29]; Stakeholders’ documents [30, 31]; Position reviews [32–36].	Systematic reviews [37]; Position reviews [38].	Systematic reviews [39]; Stakeholders’ documents [40]; Position reviews [41].
Incentives to participate		Systematic reviews [42–44].	Position reviews [28].
(b) Issues in participation processes and their effects on implementation			
General attrition rates^a^		Systematic reviews [42, 43, 45–50]; Position reviews [51, 52].	Systematic reviews [53, 54]; Position reviews [55, 56].
Participation levels, i.e., percent of those agreeing among eligible participants^a^			Systematic reviews [53, 54, 57, 58]; Position reviews [59].
Representativeness of attrition and dropout^a^		Systematic reviews [43, 47–49].	Systematic reviews [53, 60]; Position reviews [56].
Differential attrition across the program conditions/types^a^		Systematic reviews [43, 48].	Systematic reviews [53, 60].
(c) Cultural and social issues in reaching target populations			
Enhancing cultural competences of intervention/policy (creating culturally sensitive versions of materials)	Systematic reviews [61]; Stakeholders’ documents [62–66].	Systematic reviews [67].	Systematic reviews [26, 68]; Stakeholders’ documents [69]; Position reviews [70].
Domain: Efficacy			
(d) Satisfaction with implementation			
Participants’ satisfaction with implementation^a^		Systematic reviews [42, 44].	Systematic reviews [54, 71].
(e) Feasibility and acceptability			
Feasibility of implementation and acceptability of implementation among providers, stakeholders, and participants^a^		Systematic reviews [48, 72, 73].	Systematic reviews [57, 74–76]; Position reviews [55, 56, 59, 77].
Acceptability of the program among participants (e.g., acceptability of: the group size, the type of participants, interventionists’ skills)^a^	Systematic reviews [78]; Position reviews [33].	Systematic reviews [79]; Position reviews [38, 80].	Systematic reviews [57, 71].
(f) Evaluation of implementation/adoption processes (excluding evaluation of the outcomes of the program)			
Evaluation and monitoring results are disseminated to communities, stakeholders, and nationally	Stakeholders’ documents [31, 65, 66, 81].		Stakeholders’ documents [69].
Difficulty/a lack of opportunity to assess the impact of one policy separately from ancillary policies/interventions due to the increasing complexity of policies/legislations^a^	Systematic reviews [29, 61]; Position reviews [82, 83].		
Domain: Adoption			
(g) Training for implementation			
Training for implementers and disseminators (e.g., training, certifıcates, workshops, training instructions, skill development)	Stakeholders’ documents [30, 31, 64, 84]; Position reviews [35, 85].	Systematic reviews [5, 37, 42, 43, 48, 67, 72, 79, 86–90]; Position reviews: [51, 80, 91, 92]; Stakeholders’ documents [93].	Systematic reviews [26, 27, 54, 57, 71, 76, 94]; Position reviews [28, 56, 70, 95, 96]; Stakeholders’ documents [97].
Training instructions/materials for implementers	Position reviews [35].	Systematic reviews [42]; Position reviews [80, 92].	Systematic reviews [54, 76]
Regular meetings or supervision for staff to secure implementation		Systematic reviews [42, 90]; Position reviews [51, 92].	Position reviews [28].
(h) Staff expertise for implementation			
No additional expertise required for staff involved in implementation		Systematic reviews [43]; Position reviews [98].	Systematic reviews [60]; Position reviews [55].
Implementers’ skill, knowledge, and competence to implement the program correctly	Position reviews [35].	Systematic reviews [44, 89, 99, 100].	Systematic reviews [26, 27]; Stakeholders’ documents [97]; Position reviews [56].
(i) Collaboration and communication for implementation			
Collaboration between implementers; the use of methods to increase communication between implementers	Stakeholders’ documents [31, 62, 66].	Systematic reviews [44]; Stakeholders’ documents [93].	Systematic reviews [26, 27]; Position reviews [28, 96, 101].
Key political and stakeholders’ support for implementation (stakeholders identified and involved)	Stakeholders’ documents [62, 84, 102]; Position reviews [85, 103, 104].		Systematic reviews [26, 105]; Position reviews [96].
Cross-sectorial collaboration: collaboration between sectors of health, sports, food, transportation, planning and housing, green spaces, education, healthcare, and social services	Stakeholders’ documents [63, 64, 81, 84, 102]; Position reviews [34, 104, 106–110].	Position reviews [91].	Stakeholders’ documents [40, 69, 97]; Position reviews [41, 70, 95].
Involvement of multiple stakeholders at multiple levels	Stakeholders’ documents [31, 64, 84, 102]; Position reviews [35, 107].		Stakeholders’ documents [97].
Collaboration with professionals and organizations for program implementation		Systematic reviews [5, 100]	Systematic reviews [111]; Position reviews [41, 112].
Effective leadership to secure collaboration (between facilitators, institutions, and organizations involved)	Stakeholders’ documents [30, 31, 62, 64, 65, 81, 84, 102].	Systematic reviews [44, 88].	Stakeholders’ documents [97].
Synergy with other existing or operating programs	Position reviews [34, 104, 108–110, 113].	Position reviews [114].	Position reviews [41].
Securing food industry involvement/preventing and counteracting food industry resistance	Stakeholders’ documents [64]; Position reviews [35, 36, 109, 113, 115]		
(j) Community support for implementation			
Securing the involvement of local community in implementation	Systematic reviews [29]; Stakeholders’ documents [31, 84, 102, 116]; Position reviews [33, 107].		Systematic reviews [54, 71]; Stakeholders’ documents [40].
Community organizations support adoption	Stakeholders’ documents [31, 102, 116].	Systematic reviews [42, 86].	Stakeholders’ documents [40, 69]; Position reviews [28, 101].
Building relationships/networks for implementation (between implementing organizations and community organizations)	Stakeholders’ documents [31]; Position reviews [117].	Systematic reviews [100].	Systematic reviews [26].
(k) Adoption in physical environment facilitating implementation			
Maintenance or development of built and natural environment to enable policies implementation	Stakeholders’ documents [30, 62, 63, 81, 116]; Position reviews [83, 107, 110].	Systematic reviews [67].	Systematic reviews [39, 105]; Stakeholders’ documents [97].
Supportive physical environment in the community promotes implementation and adoption	Stakeholders’ documents [30, 63, 116]; Position reviews [34].	Systematic reviews [72].	Stakeholders’ documents [40].
(l) Governmental and legislative involvement			
Federal (national) government co-issues the program or is involved in program issuing	Systematic reviews [29]; Stakeholders’ documents [84, 102, 116]; Position reviews [107, 109, 115, 117].		Position reviews [41].
Legal basis/secured legal support for implementation and maintenance (e.g., fiscal, liability instruments, market environment laws)	Stakeholders’ documents [65, 84]; Position reviews [32, 106].		
Accounting for legal instruments to support implementation (existing legal instruments supporting implementation, changes in law, and legal burden for businesses)	Position reviews [32, 33, 104, 108].		
Politicians’ collaboration (negotiation with and influencing politicians and policy makers)	Position reviews [83, 108, 109, 117].		
Involvement of a local government and accounting for regional regulations	Stakeholders’ documents [63, 102]; Position reviews [33, 34, 109].		Stakeholders’ documents [40]; Position reviews [118].
Accounting for conflicting policies in adoption process^a^	Position reviews [35, 110, 115, 119].		
Domain: consistency, cost, and adaptations in Implementation			
(m) Simplicity as a factor facilitating implementation			
Simplicity of communicating and implementing the program (not too complex, not too difficult to follow)	Stakeholders’ documents [66].	Systematic reviews [44, 50, 88]; Position reviews [38, 98, 114].	Systematic reviews [71, 74].
Complexities of existing policies and their interrelations as barriers to implementation^a^	Systematic reviews [61]; Position reviews [82, 83, 108].		
(n) Accessibility for participants			
Increasing accessibility to environmental structures	Stakeholders’ documents [63, 81]; Position reviews [107]		Stakeholders’ documents [40].
Financially accessible programs (low-cost, high affordability)	Position reviews [33, 35, 107].	Systematic reviews [44].	Stakeholders’ documents [40]; Position reviews [28, 55, 112].
Barriers for accessibility in physical environment (e.g., architectural solutions as barriers to exercise; a lack of stairs)^a^	Stakeholders’ documents [63].	Systematic reviews [120], Position reviews [121].	Stakeholders’ documents [40]; Position reviews [70].
(o) Evaluating and solving time-related issues in implementation			
Lack of time in the community involved in implementation^a^	Position reviews [33].	Systematic reviews [122]; Position reviews [123].	Stakeholders’ documents [69].
Time for implementation: assessment of time needed for implementation conducted and adequate time secured	Stakeholders’ documents [62]; Position reviews [33].	Systematic reviews [44].	Stakeholders’ documents [69].
Limited time in curriculum to add new program in respective settings (e.g., schools)	Position reviews [115].	Systematic reviews [122]; Position reviews [123].	Position reviews [70].
(p) Fidelity			
Fidelity of the program (in reference to the content and the dose of the program)	Stakeholders’ documents [64].	Systematic reviews [44, 72, 79, 87, 88]; Stakeholders’ documents [124].	Position reviews [56].
Degree to which intervention is delivered as intended (compared to the protocol)		Systematic reviews [42, 46, 125]; Position reviews: [98].	
Assessment of fidelity of delivery^a^		Systematic reviews [42, 46, 48, 87].	
(q) Use of implementation theory/framework			
Use of implementation theory for implementation practice		Systematic reviews [86, 126]; Position reviews [80].	Systematic reviews [39, 76].
Use of RE-AIM framework for identification, appraisal, and synthesis of material		Systematic reviews [43, 45–47, 79, 125, 127]; Position reviews [51, 52, 98, 128]	Systematic reviews [74]; Position reviews [28].
(r) Cultural context in implementation			
Culture-sensitive implementation, addressing the needs of diverse population in their community context (social, cultural, economic, and political)	Systematic reviews [29]; Stakeholders’ documents [31, 62–66]; Position reviews [35, 83, 104].	Systematic reviews [67, 79, 86, 89]; Position reviews [80, 91, 114, 123].	Systematic reviews [26, 71, 94]; Stakeholders’ documents [69, 97]; Position reviews [70, 118].
(s) Costs and funding of implementation			
Costs of implementation analyzed (e.g., analysis of costs to deliver per person)	Position reviews [33].	Systematic reviews [42, 45, 46, 72, 79, 125].	Systematic reviews [54, 71].
Funding/resources for implementation secured and provided	Systematic reviews [61]; Stakeholders’ documents [62, 65, 84, 102, 116]; Position reviews [103, 107].	Systematic reviews [100]; Position reviews [80].	Systematic reviews [27, 71, 75]; Stakeholders’ documents [69].
Lack of/limited funding for implementation^a^	Position reviews [33, 34, 107, 117].	Systematic reviews [88, 120]; Stakeholder documents [93].	Position reviews [41].
Cost targets: low (feasible) costs of implementation, cheap resources, and affordable across settings	Stakeholders’ documents [64]; Position reviews [85].	Systematic reviews [37, 129].	Systematic reviews [74]; Position reviews [28].
Securing funds for long-term maintenance (e.g., through national government funds)	Position reviews [33].	Systematic reviews [46, 86].	Stakeholders’ documents [40].
(t) Other resources needed for delivery			
Lack of resources for implementation in organizations involved in delivery^a^	Systematic reviews [61]; Position reviews [117].	Systematic reviews [44]; Position reviews [123].	Systematic reviews [71].
Lack of resources for implementation (from sources other than involved organizations)^a^	Stakeholders’ documents [62]; Position reviews [34].	Systematic reviews [46, 100].	Position reviews [41].
(u) Delivery characteristics			
Extent to which protocol was delivered as intended/protocol adherence		Systematic reviews [45, 47, 99, 125]; Position reviews [52, 128].	Systematic reviews [71].
Consistency of delivery and evaluation/monitoring of consistency	Position reviews [35].	Systematic reviews [5, 43, 46, 48]; Position reviews [52].	Systematic reviews [60]; Position reviews [101].
Identifying the essential amount of time/number of sessions required to deliver the program	Position reviews [107].	Systematic reviews [42, 43, 48]; Position reviews [128].	Systematic reviews: [60].
Mass media involved in delivery and dissemination	Stakeholders’ documents [102]; Position reviews [35, 113, 115].	Stakeholders’ documents [93].	
Involving any available staff into the program delivery		Systematic reviews [67, 90, 126]; Position reviews [123].	
Clear identification of roles and responsibilities in implementation processes	Stakeholders’ documents [62]; Position reviews [34, 103]	Stakeholders’ documents [93]	
Delivery through various professional groups, lay health advisors, and users	Position reviews [34, 107, 109].	Systematic reviews [86, 126]. Position reviews [114, 123].	
Pilots: testing new and existing materials before delivering to the target population	Position reviews [82].	Systematic reviews [90]; Position reviews [38, 114].	Position reviews [101].
(v) Settings’ characteristics affecting delivery and implementation			
Organizational practices supporting implementation, management participation in implementation	Stakeholders’ documents [30].	Systematic reviews [44]; Position reviews [114].	Stakeholders’ documents [40, 130].
Aims and existing polices within the organization are accounted for (how does the program fit into organizational aims and existing policies?)		Systematic reviews [44].	Systematic reviews [54]; Stakeholders’ documents [40, 130].
(w) Adjustments and customizations in implementation			
Deep-structure adaptations (deep cultural and ethnic adaptations to participants, consultations with community advisors on cultural adaptations, consultation with participants)	Stakeholders’ documents [64, 66].	Systematic reviews [42, 89]; Stakeholders’ documents [124]; Position reviews [38].	
Customization of the program (to target population and local conditions)		Systematic reviews [43, 47]; Stakeholders’ documents [124]; Position reviews [51, 80].	Systematic reviews [53, 60]; Position reviews [28, 56, 70, 101].
Potential adaptations to enhance the fıt within community contexts	Stakeholders’ documents [64]	Systematic reviews [43, 47, 87]; Stakeholders’ documents [124].	Systematic reviews [53, 60]; Position reviews [70, 101]
Assessment of adaptations of the intervention/policy made during delivery^a^		Systematic reviews [72].	Systematic reviews [74, 76]; Position reviews [56].
Adoption to settings^a^		Systematic reviews [44, 47, 90].	Systematic reviews [53, 54].
(x) Planning and monitoring of implementation processes			
Plans for implementation		Systematic reviews [5]; Position reviews [92].	Systematic reviews [71, 76, 94]; Position reviews [28].
Plans for monitoring and plans for evaluation (how to increase data availability and of high quality?)	Stakeholders’ documents [62, 65]; Position reviews [32, 107].	Position reviews [92].	
Process monitoring and evaluation	Position reviews [35, 83]. Stakeholders’ documents [84].	Systematic reviews [37, 90]; Position reviews [98]; Stakeholders’ documents [93].	Systematic reviews [71]; Stakeholders’ documents [69].
Monitoring and assessment of adherence to implementation protocol/protocol fidelity		Systematic reviews [47, 131].	Stakeholders’ documents [69, 97]. Position reviews [56].
(y) Implementers’ characteristics affecting implementation			
Implementers’ expectations regarding the program and perceived control of the program^a^	Position reviews [33, 85].	Systematic reviews [44, 99, 100].	
Levels of engagement/involvement and awareness of implementers	Position reviews [106].	Systematic reviews [44, 48, 99].	Stakeholders’ documents [40].
Support needed (perceived by implementers)^a^		Systematic reviews [44, 47, 88, 100].	Systematic reviews [27, 94]; Position reviews [55].
Domain: Maintenance			
(z) Sustainability			
Institutionalization of the content of the program and its implementation (e.g., the integration into existing institutional programs)		Systematic reviews [43, 48]; Position reviews [51].	Systematic reviews [57, 60]; Position reviews: [56].
Strategies to promote long-term participation (maintenance) included			Systematic reviews [26, 132]; Position reviews [28, 55].
Building capacity to secure maintenance (training and support in organization, aiming at promotion of maintenance)	Stakeholders’ documents [31, 62, 102].		Stakeholders’ documents [130].

^a^The implementation enhancement may refer to: Identification and evaluation of the issues/problems referring to respective implementation conditions, analysis of consequences for implementations, and analysis of possible solutions for better implementation

Regarding the *Reach domain of the RE-AIM framework*, we identified 29 potential implementation conditions (see Additional file 1), with eight (27.6 %) reaching the threshold for sufficient support. The implementation conditions which met the threshold are listed in Table 1. They were grouped into three distinct categories: strategies facilitating recruitment processes (*n* = 3), issues in participation processes and their effects on implementation (*n* = 4), and cultural and social issues in reaching target populations (*n* = 1). All conditions were generic.

The analysis of original documents yielded 19 potential implementation conditions referring to the *Efficacy domain of the RE-AIM framework* (Additional file 1). Only five conditions (26.3 %) met the sufficient support threshold (see Table 1). These implementation conditions were grouped into three categories: satisfaction with implementation (*n* = 1), feasibility and acceptability (*n* = 2), evaluation of implementation/adoption processes (excluding evaluation of the outcomes of the program) (*n* = 2). The majority of implementation conditions from this domain (*n* = 4) were generic, but one condition was specific for policies only. It referred to a difficulty or lack of opportunity to assess the impact of one policy separately from ancillary policies/interventions due to the increasing complexity of policies/legislations.

We identified 94 potential implementation conditions referring to the *Adoption domain of the RE-AIM framework* (Additional file 1) and 24 (25 %) met the threshold for sufficient support (Table 1). The implementation conditions capturing adoption processes were grouped into six broader categories: training for implementation (*n* = 3), staff expertise for implementation (*n* = 2), collaboration and communication for implementation (*n* = 7), community support for implementation (*n* = 3), adoption in physical environment facilitating implementation (*n* = 2), and the governmental and legislative involvement (*n* = 6). Although the majority (*n* = 19) of the implementation conditions were generic, five conditions referred to policies only: securing food industry involvement/preventing and counteracting food industry resistance, legal basis/secured legal support for implementation and maintenance (e.g., fiscal, liability instruments, market environment laws), accounting for legal instruments to support implementation (existing legal instruments supporting implementation, changes in law, and legal burden for businesses), politicians’ collaboration (negotiation with and influencing politicians and policy makers), and accounting for conflicting policies in adoption process.

The analysis yielded 149 potential conditions referring to *the domain of the RE-AIM framework addressing to the consistency, costs, and adaptations in Implementation* (Additional file 1). Among these implementation conditions 43 (28.8 %) met the sufficient support threshold (Table 1). They were grouped into 13 categories: simplicity as a factor facilitating implementation (*n* = 2), accessibility for participants (*n* = 3), evaluating and solving time-related issues in implementation (*n* = 3), fidelity (*n* = 3), the use of implementation theory/framework (*n* = 2), cultural contexts in implementation (*n* = 1), cost and funding of implementation (*n* = 5), other resources needed for delivery (*n* = 2), delivery characteristics (*n* = 8), settings’ characteristics affecting delivery and implementation (*n* = 2), adjustments and customizations in implementation (*n* = 5), planning and monitoring of implementation processes (*n* = 4), and implementers’ characteristics affecting implementation (*n* = 3). Although the majority of implementation conditions were generic (*n* = 39), one was specific for policies: complexities of existing policies and their interrelations as barriers to implementation and three conditions were specific for interventions: the degree to which an intervention is delivered as intended (compared to the protocol), assessment of fidelity of delivery, and involving any available staff into the program delivery.

Finally, we elicited 21 potential conditions referring to *the Maintenance domain of the RE-AIM framework* (Additional file 1). Only three (14.3 %) of these implementation conditions were supported by at least four documents and therefore considered as obtaining sufficient support (Table 1). The three conditions were organized into one category, referring to sustainability. The implementation conditions representing maintenance domain were generic i.e., they referred to both interventions and policies.

In summary, data synthesis yielded 83 implementation conditions, which may be allocated to the domains of the RE-AIM framework. The list of implementation conditions was combined into a checklist (Table 2), which may be used for developing practice and reporting research on interventions and policies.

**Table 2:** The checklist of implementation conditions for interventions and policies aiming at nutrition behavior, sedentary behavior, and physical activity change

No.	Implementation domain	Page no. (in a report or protocol of evaluated intervention/policy)
	Implementation condition	
	Implementation domain: Reach	
1a	Resources/strategies for implementers helping them to invite and follow-up participants	
2a	Awareness raising (strategies to raise awareness of dietary behavior, physical activity, sedentary behaviors, as well as interventions and policies) to help implementers to invite participants	
3a	Incentives to participate	
4b	General attrition rates^a^	
5b	Participation levels, i.e., percent of those agreeing among eligible participants^a^	
6b	Representativeness of attrition and dropout^a^	
7b	Differential attrition across the program conditions/types^a^	
8c	Enhancing cultural competences of intervention/policy (creating culturally sensitive versions of materials)	
	Implementation domain: Efficacy	
9d	Participants’ satisfaction with implementation^a^	
10e	Feasibility of implementation and acceptability of implementation among providers, stakeholders, and participants^a^	
11e	Acceptability of the program among participants (e.g., acceptability of the group size, the type of participants, interventionists’ skills)^a^	
12f	Evaluation and monitoring results are disseminated to communities, stakeholders, and nationally	
13f	Difficulty/a lack of opportunity to assess the impact of one policy separately from ancillary policies/interventions due to the increasing complexity of policies/legislations^c,a^	
	Implementation domain: Adoption	
14g	Training for implementers and disseminators (e.g. training, certifıcates, workshops, training instructions)	
15g	Training instructions/materials for implementers	
16g	Regular meetings or supervision for staff to secure implementation	
17h	No additional expertise required for staff involved in implementation	
18h	Implementers’ skill, knowledge, and competence to implement the program correctly	
19i	Collaboration between implementers; the use of methods to increase communication between implementers	
20i	Key political and stakeholders’ support for implementation (stakeholders identified and involved)	
21i	Cross-sectorial collaboration: collaboration between sectors of health, sports, food, transportation, planning and housing, green spaces, education, healthcare, and social services	
22i	Involvement of multiple stakeholders at multiple levels	
23i	Collaboration with professionals and organizations for program implementation	
24i	Effective leadership to secure collaboration (between facilitators, institutions, and organizations involved)	
25i	Synergy with other existing or operating programs	
26i	Securing food industry involvement/preventing and counteracting food industry resistance^c^	
27j	Securing the involvement of local community in implementation	
28j	Community organizations support adoption	
29j	Building relationships/networks for implementation (between implementing organizations and community organizations)	
30k	Maintenance or development of built and natural environment to enable policies implementation	
31k	Supportive physical environment in the community promotes implementation and adoption	
32l	Federal (national) government co-issues the program or is involved in program issuing	
33l	Legal basis/secured legal support for implementation and maintenance (e.g. fiscal, liability instruments, market environment laws)^c^	
34l	Accounting for legal instruments to support implementation (existing legal instruments supporting implementation, changes in law, and legal burden for businesses)^c^	
35l	Politicians’ collaboration (negotiation with and influencing politicians and policy makers)^c^	
36l	Involvement of a local government and accounting for regional regulations	
37l	Accounting for conflicting policies in adoption process^c,a^	
	Implementation domain: Consistency, cost, and adaptations in implementation	
38m	Simplicity of communicating the program (not too complex, not too difficult to follow)	
39m	Complexities of existing policies and their interrelations as barriers to implementation^c,a^	
40n	Increasing accessibility to environmental structures	
41n	Financially accessible programs (low-cost, high affordability)	
42n	Barriers for accessibility in physical environment (e.g., architectural solutions as barriers to exercise; a lack of stairs)^a^	
43o	Lack of time in the community involved in implementation^a^	
44o	Time for implementation: assessment of time needed for implementation conducted and adequate time secured	
45o	Limited time in curriculum to add new program in respective setting (e.g., schools)	
46p	Fidelity of the program (in reference to the content and the dose of the program)	
47p	Degree to which intervention is delivered as intended (compared to the protocol)^b^	
48p	Assessment of fidelity of delivery^b,a^	
49q	Use of implementation theory for implementation practice	
50q	Use of RE-AIM framework for identification, appraisal, and synthesis of material	
51r	Culture-sensitive implementation, addressing the needs of diverse population in their community context (social, cultural, economic, and political)	
52s	Costs of implementation analyzed (e.g., analysis of costs to deliver per person)	
53s	Funding/resources for implementation secured and provided	
54s	Lack of/limited funding for implementation^a^	
55s	Cost targets: low (feasible) costs of implementation, cheap resources, and affordable across settings	
56s	Securing funds for long-term maintenance (e.g., through national government funds)	
57t	Lack of resources for implementation in organizations involved in delivery^a^	
58t	Lack of resources for implementation (from sources other than organizations involved)^a^	
59u	Extent to which protocol was delivered as intended/protocol adherence	
60u	Consistency of delivery and evaluation/monitoring of consistency	
61u	Identifying the essential amount of time/number of sessions required to deliver the program	
62u	Mass media involved in delivery and dissemination	
63u	Involving any available staff into the program delivery^b^	
64u	Clear identification of roles and responsibilities in implementation processes	
65u	Delivery through various professional groups, lay health advisors, and users	
66u	Pilots: testing new and existing materials before delivering to the target population	
67v	Organizational practices supporting implementation, management participation in implementation	
68v	Aims and existing polices within the organization are accounted for (does the program fit into organizational aims and existing policies?)	
69w	Deep-structure adaptations (e.g., deep cultural and ethnic adaptations to participants, consultations with community advisors on cultural adaptations, consultation with participants)	
70w	Customization of the program (to target population and local conditions)	
71w	Potential adaptations to enhance the fıt within community contexts	
72w	Assessment of adaptations of the intervention/policy made during delivery^a^	
73w	Adoption to settings^a^	
74w	Plans for implementation	
75w	Plans for monitoring and plans for evaluation (how to increase data availability and of high quality?)	
76w	Process monitoring and evaluation	
77w	Monitoring and assessment of adherence to implementation protocol/protocol fidelity	
78x	Implementers’ expectations regarding the program and perceived control of the program^a^	
79x	Levels of engagement/involvement and awareness of implementers	
80x	Support needed (perceived by implementers)^a^	
	Implementation domain: Maintenance	
81z	Institutionalization of the program content and its implementation (e.g., the integration into existing institutional programs)	
82z	Strategies to promote long-term participation (maintenance) included	
83z	Building capacity to secure maintenance (training and support in organization, aiming at promotion of maintenance)	

‘a’ to ‘z’ represent 25 implementation categories (for categories see Table 1); Page no.- if the list is used for reporting on or evaluating interventions/policies, please indicate the page in the original report/protocol where the characteristic is addressed

^a^The implementation enhancement may refer to: Identification and evaluation of the issues/problems referring to respective implementation conditions, analysis of consequences for implementations, and analysis of possible solutions for better implementation

^b^characteristic identified only documents referring to interventions

^c^characteristic identified only in documents referring to policy

## Discussion

This study provides an insight into conditions important for implementation of interventions and policies targeting healthy diet, PA, and SB in various populations. We identified 83 conditions of which the majority ( 87.9 %) were supported by documents addressing both interventions and policies.

This study aimed to contribute to the development of an evidence-based list of implementation conditions (Table 2), which provide a comprehensive overview and operationalize aspects of implementation which may contribute to the overall effectiveness of interventions and policies promoting healthy diet and PA. Other lists of implementation conditions illustrate certain frameworks or models by unsystematic review of existing research [16, 17]. These lists are usually generic in terms of behavior (i.e., assumed to be feasible for any health promoting actions). In contrast, our list was developed by systematically reviewing empirical evidence obtained in systematic reviews, documents of major stakeholders, and evidence-based positon papers. Therefore, our list represents - more so than earlier attempts - an accumulation of evidence, guidelines and practice in implementation of policies and interventions promoting healthy diet and PA.

Our efforts to identify characteristics which are evidence and practice-based were undertaken in a response to concerns of practitioners and researchers indicating difficulties in the processes of translating laboratory-based interventions and polices into real-life contexts [17]. The replications and transfer of successful interventions and policies to other settings, communities, and cultures may depend on availability of detailed protocols of implementation conditions. The ‘adoptions, adaptations, and actions’ used in the implementation processes are likely to influence the obtained effects of policies and interventions ([17], p. 600). Thorough reporting of the content of policies and interventions and implementation conditions is essential for identification of the components that are responsible for the success of interventions or policies [7]. Therefore, the protocols of interventions and polices should thoroughly discuss details of implementation conditions, which may be in part responsible for success (or failure) of interventions or policies.

The choice of RE-AIM [15] as the guiding framework results in highlighting certain domains of implementation, such as Reach. Other frameworks or approaches may stress other implementation domains, such as fidelity in a more robust fashion [6, 7]. Any comparisons made between the programs and evaluating their outcomes are based on assumptions that the program was delivered as originally intended. The present study applied RE-AIM merely for the purpose of organizing the findings. The results should not be interpreted as verifying RE-AIM or pointing to the importance of certain domains. The findings do not assume a hierarchy of domains, broader thematic characteristics or specific implementation conditions. Further research is necessary to elicit which implementation conditions are the strongest determinants of success of intervention/policies.

Our study resulted in a development of an evidence-based checklist of implementation conditions (Table 2), which may guide the planning, development, and reporting of interventions and policies addressing diet, PA, and SB. This list includes the implementation conditions supported in our study and a column (at a right hand side) for the researcher or practitioner to indicate if a condition was addressed by the intervention/policy being evaluated. Our checklist also facilitates follow-up analyses, for example, one has the space to note where the condition was addressed in the protocols or reports of intervention/policies.

Due to its evidence and practice-based character it may be useful for researchers, practitioners, and stakeholders. Researchers may use the list as a set of factors which need to be taken into account when developing and evaluating real-world interventions or policies. The list can be treated as an evidence-based set of conditions which are likely to facilitate implementation and increase the likelihood of a success of the intervention. The use of the list by researchers would improve reporting on interventions/policies which have been already implemented.

This evidence-based list may be used by stakeholders, practitioners and specialist responsible for developing and evaluating large-scale public health interventions and policies. It may be applied as a checklist for identifying implementation conditions which: (1) were not accounted for while developing the program, (2) were emphasized when the policy/intervention was applied, and (3) were not considered when the intervention/policy was developed and thus the likelihood of successful implementation may be reduced.

Substantial effort has been recently devoted to promote detailed reporting of the content of interventions and policies [8], yet the content of interventions and policies is implemented in specific contexts [16]. To date, discussion on the role of “decontextualizing” interventions and policies has focused mainly on translating laboratory-based evidence to real-life settings [16]. More thorough reporting and investigation of implementation conditions could be applied in small-scale efficacy research or preliminary tests of intervention/policy effects to identify critical implementation conditions which facilitate or hinder the effects of specific behavior change techniques. Furthermore, researchers should more often consider assessing implementation conditions through evaluating policies as natural experiments, taking place in specific socio-cultural contexts. This approach could foster research on policy implementation and therefore allow for accumulating empirical evidence for eliciting the most essential implementation conditions.

As some existing implementation frameworks and models account for policies only, we investigated implementation conditions which may be policy-specific. The results indicated that policy-specific implementation conditions share some common themes. First, they refer to increasing complexities of coexisting policies and legal instruments, which operate in a parallel manner, to facilitate or hinder the implementation of a new policy. Second, the policy-specific implementation conditions stress a need to account for legal instruments (existing and newly developed for policy implementation) and industry involvement at various stages of implementation. These conditions may reflect the differences between interventions and polices in the scale of the actions (i.e., policies are operating at regional or wider scale). In particular, the implementation of actions operating on a broader scale (e.g. regional, national) may be more dependent on involvement of industry and legal acts supporting initiation and maintenance of population-targeting policies.

Our study has several limitations. First, we used an arbitrary criterion for distinguishing between policies and interventions based on a WHO definition [4], referring to the scale of operation (i.e. international versus local). However, there are many cases of policies developed and enacted on a local level, and there are examples of large scale interventions that are regional and national in scope. In consequence, several health promotion programs were arbitrarily categorized as either promotion or policies, although they might have characteristics of both. The findings indicating a limited number of conditions which were either policy-specific or intervention-specific may be the consequence of applied categorization. The proposed list is based on a review of documents which vary in terms of the methodology used to elicit implementation conditions and in terms of the quality of the methods applied. Future research is warranted to investigate how the methods of obtaining data or the type of data sources affect the content of the list of implementation conditions. In applying umbrella review strategies we did not account for the most recent research. As new evidence accumulates each year, the proposed list of implementation conditions will require a regular update and verification. Compared to other proposals listing relevant implementation conditions in policies and interventions, our efforts were confined to actions referring to healthy diet, PA, and SB. However, we did not analyze if the conditions may be behavior-specific or if they may be only relevant in specific populations (e.g. children). The categorization of implementation conditions was conducted using definitions or brief descriptions of these conditions, provided in reviews or stakeholders documents. Therefore, our work represents a second-level categorization, relying on the quality of the categorization processes used in the analyzed documents. Finally, the applied inclusion threshold was based on an arbitrary cut-off point of the upper quartile for the number of documents supporting a characteristic in either the systematic reviews, position papers or stakeholder documents). Although similar quartile-based thresholds are used in health promotion research [9, 25], this threshold does not reflect the quality of obtained evidence, but rather the focus of research conducted so far. The change of the inclusion threshold (e.g., to the upper 15 %) would result in a reduction of the number of implementation characteristics to 58. However, such change of the threshold could result in bias towards excluding conditions which are investigated less often. In particular, a half of the policy-specific implementation conditions would be excluded, if such threshold pertained. Future research is needed to conduct a thorough sensitivity analysis which would take into account the quality of the documents included and the types of the analyzed documents). In summary, all conclusions should be treated with caution and the list of characteristics may change with future research.

## Conclusions

In conclusion, this review resulted in developing a list of 83 conditions important for successful implementation of interventions and policies. The list is based on accumulating research evidence and the position of major stakeholders responsible for developing guidelines in health promotion. The list may enhance the implementation of interventions and policies which pursue identification of most successful actions aimed at promoting healthy diet, PA, and a reduction of SB in various populations. It may facilitate future research in that the list may be seen as a point of departure for further syntheses. The list might be shortened if future research demonstrates a lack of relevance of some implementation conditions used in the context of policies and interventions addressing diet, PA, and SB. Until then, this broad list has a potential to inspire accumulating more detailed data and guide the process of application of polices and interventions across contexts.

## Additional file

Additional file 1:
**PRISMA checklist, quality evaluation criteria for stakeholders’ documents, descriptive data for all reviewed documents and the list of 312 elicited characteristics (with supporting documents).** Description of the file: Additional file includes: (a) the PRISMA checklist; (b) the quality evaluation criteria for stakeholders’ documents (Methodological Quality Checklist for Stakeholders’ Documents and Position Papers (MQC-SP); (c) descriptive data retrieved from systematic reviews, stakeholder documents and position review papers included into the umbrella review; (d) the list of 312 implementation characteristics and references to the documents supporting the characteristics. (DOCX 206 kb)

## Abbreviations

DEDIPAC: The determinants of diet and physical activity knowledge hub
MQC: Methodological quality checklist
MQC-SP: Methodological quality checklist – for stakeholder documents and position papers
