# Supplementary material for: Implementation conditions for diet and physical activity interventions and policies: an umbrella review
Source: BMC Public Health. 2015 Dec 17;15:1250. doi: 10.1186/s12889-015-2585-5 (PMC4683715; doi:10.1186/s12889-015-2585-5)
Supplement: Additional file 1: — PRISMA checklist, quality evaluation criteria for stakeholders’ documents, descriptive data for all reviewed documents and the list of 312 elicited characteristics (with supporting documents). Description of the file: Additional file includes: (a) the PRISMA checklist; (b) the quality evaluation criteria for stakeholders’ documents (Methodological Quality Checklist for Stakeholders’ Documents and Position Papers (MQC-SP); (c) descriptive data retrieved from systematic reviews, stakeholder documents and position review papers included into the umbrella review; (d) the list of 312 implementation characteristics and references to the documents supporting the characteristics. (DOCX 206 kb) [file 12889_2015_2585_MOESM1_ESM.docx]

**Additional file 1**

*Table 1. PRISMA Checklist*

| **Section/topic** | **#** | **Checklist item** | **Reported on page #** |
| --- | --- | --- | --- |
| **TITLE** | | |  |
| Title | 1 | Identify the report as a systematic review, meta-analysis, or both. | 1 |
| **ABSTRACT** | | |  |
| Structured summary | 2 | Provide a structured summary including, as applicable: background; objectives; data sources; study eligibility criteria, participants, and interventions; study appraisal and synthesis methods; results; limitations; conclusions and implications of key findings; systematic review registration number. | 3 |
| **INTRODUCTION** | | |  |
| Rationale | 3 | Describe the rationale for the review in the context of what is already known. | 5, 6, 7, 8 |
| Objectives | 4 | Provide an explicit statement of questions being addressed with reference to participants, interventions, comparisons, outcomes, and study design (PICOS). | 8, 9 |
| **METHODS** | | |  |
| Protocol and registration | 5 | Indicate if a review protocol exists, if and where it can be accessed (e.g., Web address), and, if available, provide registration information including registration number. | 9 |
| Eligibility criteria | 6 | Specify study characteristics (e.g., PICOS, length of follow-up) and report characteristics (e.g., years considered, language, publication status) used as criteria for eligibility, giving rationale. | 9, 10, 11, 12, 13; see also Additional file 1 (Table 3) |
| Information sources | 7 | Describe all information sources (e.g., databases with dates of coverage, contact with study authors to identify additional studies) in the search and date last searched. | 10, 13 |
| Search | 8 | Present full electronic search strategy for at least one database, including any limits used, such that it could be repeated. | 10, 11, 13 |
| Study selection | 9 | State the process for selecting studies (i.e., screening, eligibility, included in systematic review, and, if applicable, included in the meta-analysis). | 10, 11, 12, 13, 14 |
| Data collection process | 10 | Describe method of data extraction from reports (e.g., piloted forms, independently, in duplicate) and any processes for obtaining and confirming data from investigators. | 14, 15 |
| Data items | 11 | List and define all variables for which data were sought (e.g., PICOS, funding sources) and any assumptions and simplifications made. | 14, 15, 16; see also Additional file (Tables 3 and 4) |
| Risk of bias in individual studies | 12 | Describe methods used for assessing risk of bias of individual studies (including specification of whether this was done at the study or outcome level), and how this information is to be used in any data synthesis. | 11, 12, 14; see also Additional file 1 (Table 2; Table 3 – the last column) |
| Summary measures | 13 | State the principal summary measures (e.g., risk ratio, difference in means). | 16, 17 |
| Synthesis of results | 14 | Describe the methods of handling data and combining results of studies, if done, including measures of consistency (e.g., I^2^) for each meta-analysis. | 14, 16, 17 |

*Table 2. Methodological Quality Checklist for Stakeholders’ Documents and Position Papers (MQC-SP)*

| Criteria and their description | Scoring |
| --- | --- |
| **1. Is there a major stakeholder involved?**  - The document is developed/endorsed by (1) a nation-wide or international organization which is issuing recommendations and guidelines which are used in clinical practice; or (2) an interdisciplinary or cross-country consortium aiming at providing progress in the discipline/practice for respective behavior | 0 (no) or 1 (yes) |
| **2. Is there a well-defined aim?**  – The document specifies the aim of the paper, target population, the type of actions and their breadth (e.g., changes in physical environment, any school-based interventions and policies) and the type of relevant behavior | 0 (no) or 1 (yes) |
| **3. Is there a robust methodology?**  – The method should list the sources used to obtain *comprehensive and heterogeneous* data, such as literature review and analysis of several examples of interventions/policies, and “grey literature” or unpublished documents | 0 (no) or 1 (yes) |
| **4. Quality evaluation of analyzed material applied?**  – The document refers to the quality evaluation of the included material and/or refers to quality evaluation methods or measures | 0 (no) or 1 (yes) |
| **5. Have the included material been appropriately synthesized?**  – The synthesis of analyzed material addresses the heterogeneity of analyzed data; provides specific conclusions; conclusions are supported by analyzed material; the key constructs are clearly operationalized | 0 (no) or 1 (yes) |
| **6. Has more than one stakeholder/author been involved at the process?**  – To minimize bias, conclusions were based on involvement and consensus achieved by at least two stakeholders/ multiple researchers from different organizations | 0 (no) or 1 (yes) |
| **Total score: low = 0-3, moderate: 4-5; high 6** | 0-6 |

*Table 3. Description of systematic reviews, stakeholder documents and position review papers included into the umbrella review.*

| Author, year | The type of document | No. original studies | Original study design | Population | Behavior | Policy or Intervention | Quality  score |
| --- | --- | --- | --- | --- | --- | --- | --- |
| American Dietetic Association 2003 | 3 | - | - | Children | Diet and PA | Both | 4 |
| Akers 2010 | 1 | 19 | RCT | Adults | PA and Diet | Intervention | 5 |
| Antikainen 2011 | 3 | - | - | Vulnerable population | PA | Intervention | 3 |
| Ayliffe 2010 | 1 | 63 | RCT, Cluster RT, CT, Prospective cohort studies | Children | Diet and PA | Both | 5 |
| Baker 2011 | 1 | 25 | RCT, Cluster RCT, quasi-experimental, interrupted time-series (ITS) studies, prospective controlled cohort studies | General population | PA | Both | 7 |
| Bellew 2008 | 3 | - | - | General population | PA | Policy | 5 |
| Beets 2009 | 1 | 13 | RCT, quasi-experimental | Children and adolescents | PA and SB | Intervention | 5 |
| Benjamin 2014 | 1 | 18 | RCT, qualitative studies | Adults (older, living in LTC) | PA | Intervention | 5 |
| Besculides 2008 | 3 | - | - | Adults (women) | Diet and PA | Both | 5 |
| Birch 2011 (IOM 2011) | 2 | - | - | Children | PA and Diet | Policy | 5 |
| Blackman 2013 | 1 | 15 | RTC, quasi-experimental | General population | PA | Intervention | 4 |
| Brennan 2014 | 1 | 396 | RCT, observational, cross-sectional | Children | Diet and PA | Both | 5 |
| Bundy 2012 | 3 | - | - | Children | Diet | Policy | 4 |
| Cambon 2012 | 3 | - | - | General population | PA and Diet | Intervention | 4 |
| Capacci 2012 | 3 | - | - | General populations | Diet | Policy | 5 |
| Caraher 2007 | 3 | - | - | General populations | Diet | Policy | 4 |
| Cardona-Morrell 2010 | 1 | 12 | RCT, cluster RCT, observational, before-after designs with a control group | Adults (in diabetes prevention context) | PA and Diet | Intervention | 4 |
| Carlsson 2008 | 3 | - | - | Children | Diet | Policy | 3 |
| Carroll 2011 | 1 | 38 | RCT, CT, quasi-experimental, cluster CT | Adults (primary care) | PA | Both | 5 |
| CDC 2011 | 2 | - | - | Children | Diet and PA | Policy | 6 |
| Christiansen 2014 | 3 | - | - | General population | PA | Policy | 5 |
| Craig 2011 | 3 | - | - | General population | PA | Policy | 4 |
| Crammond 2013 | 3 | - | - | General population | Diet | Policy | 5 |
| Diepeveen 2013 | 1 | 200 | multicriterion  mapping studies, CT, non-experimental | General population | PA and Diet | Policy | 4 |
| Doak 2006 | 1 | 25 | RCT, CT | Children and adolescents | PA and Diet | Intervention | 4 |
| Dzewaltowski, 2004 | 3 | - | - | General population | PA and Diet | Intervention | 4 |
| Eakin 2002 | 1 | 10 | RCT, quasi -experimental | Vulnerable populations | Diet and PA | Intervention | 3 |
| Eakin, 2005 | 3 | - | - | General population | PA | Intervention | 3 |
| Fleury 2009 | 1 | 20 | RCT, observational, quasi-experimental | Vulnerable populations | PA and Diet | Intervention | 5 |
| Flynn 2006 | 1 | 147 | RCT, CT, quasi-experimental, cohort, observational | Children | Diet and PA | Both | 7 |
| Fransen 2012 | 3 | - | - | General population | Diet and PA | Both | 4 |
| Freudenberg 2010 | 3 | - | - | Children | PA and Diet | Policy | 3 |
| Galaviz 2014 | 1 | 46 | Experimental, quasi-experimental | General population | PA | Intervention | 4 |
| Gaziano 2007 | 3 | - | - | Vulnerable population | PA and Diet | Policy | 3 |
| Geaney 2013 | 1 | 6 | RCT, CT, cluster RCT, quasi-experimental observational | Adults (at workplace) | Diet | Both | 6 |
| Ghisi 2014 | 1 | 42 | RCT, CT, quasi-experimental, observational, cohort, cross-sectional, correlational-longitudinal | Adults (with chronic disease) | Diet and PA | Intervention | 6 |
| Gillison 2012 | 3 | - | - | General population | Diet and PA | Both | 5 |
| Glasgow 2007 | 3 | - | - | General population | Diet and PA | Both | 4 |
| Glickman 2012 (IOM 2012) | 2 | - | - | General population | PA and Diet | Policy | 5 |
| Goode 2012 | 1 | 25 | RCT, CT, observational | General population | Diet and PA | Intervention | 6 |
| Goodwin 2011 | 1 |  | Non RCT, cross-sectional, cohort, surveys, process evaluation, observational | Adults (older in context of fall prevention) | PA | Intervention | 6 |
| Haughton 2012 | 3 | - | - | Adults (older) | Diet | Both | 4 |
| Hearn 2008 | 1 | 45 | RCT, CT, quasi-experimental, observational | Children | Diet and PA | Both | 4 |
| Heath 2006 | 1 | 19 | Any quantitative | General population | PA | Both | 4 |
| Heath 2012 | 1 | 100 | Reviews (systematic and non-systematic) | General population | PA | Intervention | 5 |
| Hoehner 2013 | 1 | 19 | RCT, CT, quasi-experimental | General population | PA | Both | 7 |
| Hoelscher 2013 | 3 | - | - | Children | Diet and PA | Both | 6 |
| Huijg 2014 | 1 | 59 | Cross-sectional, RCT, qualitative, observational, quasi-experimental | General population | PA | Intervention | 4 |
| Ickes 2013 | 1 | 13 | RCT, quasi-experimental, observational | Children and adolescents | PA | Intervention | 4 |
| Kahn 2002 | 3 | - | - | General population | PA | Both | 5 |
| Keller 2007 | 3 | - | - | General population | Diet | Policy | 3 |
| Khan 2009 | 3 | - | - | Children and adolescents | Diet and PA | Both | 6 |
| King 2011 | 3 | - | - | General population | Diet and PA | Both | 4 |
| Klesges 2008 | 1 | 19 | RCT, CT | Children and adolescents | Diet and PA | Both | 5 |
| Klesges 2012 | 1 | 77 | RCT, CT | Children and adolescents (with chronic disease) | PA and diet | Both | 5 |
| Kohl 2013 | 1 | 41 | Systematic reviews | Adults | Diet and PA | Intervention | 6 |
| Koplan 2005 (IOM 2005) | 2 | - | - | Children and adolescents | PA and Diet | Both | 5 |
| Kumanyika 2010 (IOM 2010) | 2 | - | - | General population | Diet and PA | Intervention | 6 |
| Langford 2014 | 1 | 67 | cluster RCT | Children and adolescents | Diet and PA | Intervention | 7 |
| Larson 2011 | 3 | - | - | Children | Diet and PA | Both |  |
| Laws 2012 | 1 | 31 | RCT, cluster RCT, quasi-experimental | Adults | PA and Diet | Intervention | 4 |
| Leung 2012 | 1 | 12 | RCT | Children, adolescents | SB, PA and Diet | Intervention | 5 |
| Lombard 2009 | 1 | 9 | RCT, CT | Adults | Diet and PA | Both | 5 |
| Matthews 2014 | 1 | 12 | RCT, Process Evaluation, Longitudinal, descriptive report | Adults (chronic disease) | PA and Diet | Intervention | 6 |
| McMahon 2012 | 1 | 46 | RCT, quasi-experimental | Adults (older, with fall risk) | PA | Intervention | 4 |
| Naylor 2008 | 3 | - | - | Children and adolescents | PA | Intervention | 3 |
| NICE 2008 | 2 | - | - | Adults (at workplace) | PA | Both | 4 |
| NICE 2009 | 2 | - | - | Children and adolescents | PA | Both | 4 |
| NICE 2012 | 2 | - | - | General population | PA and Diet | Policy | 4 |
| Niebylski 2014 | 1 | 34 | RCT and prospective and retrospective non randomized food procurement interventions | General population | Diet | Policy | 5 |
| Nierkens 2013 | 1 | 17 | RCT, quasi-experimental | Vulnerable population | PA and Diet | Intervention | 5 |
| McNeil 2006 | 3 | - | - | General population | Diet and PA | Both | 4 |
| Pérez-Ferrer 2010 | 3 | - | - | General population | Diet | Policy | 3 |
| Pratt 2008 | 3 | - | - | Children | Diet and PA | Both | 4 |
| Rabin 2010 | 1 | 16 | Cluster RT, cross-sectional, observational | General population | PA an Diet | Intervention | 6 |
| Ramanathan 2008 | 3 | - | - | Children | PA and Diet | Policy | 3 |
| Ribeiro 2010 | 3 | - | - | Children | PA | Intervention | 3 |
| Robertson 2012 | 1 | 8 | RCT | Adults (chronic diseases) | PA | Intervention | 6 |
| Robertson-Wilson 2012 | 1 | 13 | Cross-sectional studies | Children and youth | PA | Policy |  |
| Rütten 2013 | 3 | - | - | General population | PA | Policy | 3 |
| Sahay 2006 | 1 | 15 | RCT, CT, quasi-experimental | General population (in the context of cancer prevention) | Diet | Both | 5 |
| Shill 2012a | 3 | - | - | General population | Diet | Policy | 3 |
| Shill 2012b | 3 | - | - | General population | PA | Policy | 3 |
| Sims 2006 (NARI 2006) | 2 | - | - | Adults (older) | PA | Policy | 6 |
| Soler 2010 | 3 | - | - | Adults | PA | Intervention | 3 |
| Sorensen 2004 | 3 | - | - | Adults (at workplace) | Diet | Intervention | 3 |
| Stallings 2010 (IOM 2010) | 2 | - | - | Children | Diet | Policy | 5 |
| Stockley 2001 | 3 | - | - | General population | Diet | Both | 4 |
| Sumlin 2012 | 1 | 15 | RCT, quasi-experimental | Adults (women with chronic disease) | Diet | Both | 4 |
| Summerbell 2012 | 3 | - | - | Children | PA, SB and Diet | Intervention | 5 |
| Swedish National Institute for Public Health on behalf of the European Commission (EC 2006) | 2 | - | - | Adults (older) | PA and Diet | Intervention | 5 |
| Taylor 2013 | 3 | - | - | Children | Diet and PA | Intervention | 3 |
| Teufel-Shone 2009 | 1 | 64 | Not stated | Vulnerable population | PA | Both | 4 |
| Thow 2011 | 3 | - | - | General population | Diet | Policy | 4 |
| Tremblay 2012 | 3 | - | - | Children | PA and SB | Policy | 3 |
| Trudnak 2012 | 3 | - | - | Children | Diet and PA | Both | 4 |
| van de Vijver 2012 | 1 | 26 | RCT, quasi-experimental, cross-sectional surveys | Vulnerable population | Diet and PA | Intervention | 6 |
| van Grieken 2012 | 1 | 34 | RCT, CT | Children and adolescents | SB | Intervention | 7 |
| Verstraeten 2012 | 1 | 22 | RCT, cluster RCT, cluster RT, quasi experimental, observational | Vulnerable population | Diet and PA | Intervention | 6 |
| Vuillemin 2011 | 1 | 33 | RCT, CT, cluster CT, observational, quasi-experimental | Adults (at workplace) | PA | Intervention | 7 |
| Wang 2012 | 1 | 19 | RCT, CT, observational | Children and adolescents | Diet | Both | 6 |
| Waters 2011 | 1 | 55 | RCT, CT | Children and adolescents | PA and Diet | Both | 7 |
| Watts 2011 | 3 | - | - | General population | Diet | Intervention | 3 |
| White 2009 | 3 | - | - | Adults (breast cancer survivors) | PA | Intervention | 3 |
| Wierenga 2013 | 1 | 22 | RCT, CT | Adults (at workplace) | PA and Diet | Intervention | 7 |
| Wilding 2013 | 1 | 42 | RCT, CT, systematic reviews | Adults (older) | PA | Both | 4 |
| WHO Europe 2007a | 2 | - | - | General population | Diet and PA | Both | 6 |
| WHO Europe 2007b | 2 | - | - | General population | PA | Policy | 5 |
| WHO 2007 | 2 | - | - | General population | PA | Policy | 3 |
| WHO 2008 | 2 | - | - | Children | Diet and PA | Policy | 4 |
| WHO 2010 | 2 | - | - | General population | PA | Policy | 4 |
| WHO 2013 | 2 | - | - | General population | Diet | Policy | 3 |

*Note*: Types of documents: 1 - systematic reviews, 2 - stakeholder documents, 3 - position review papers; PA – physical activity; RCT – randomized controlled trail; CT – controlled trial; observational: studies with pre and post test for intervention group (no control group); quasi experimental – no full evaluation of pre and post tested in both control and intervention group(s); chronic diseases: obesity, cardiovascular diseases, neurological diseases, depression, diabetes, cancer; Vulnerable population - ethnic minorities, various population, indigenous population, underserved population, low and middle income population; Not stated - number of participants and study design is not available; Quality – total scores for MCQ (systematic reviews) or MCQ-SP (for stakeholders’ documents and position review papers).

*Table 4. 312 Implementation characteristics and their documentation*

| ***RE-AIM domain***  *Characteristics category*  Implementation Characteristics |  | | Behavior | | | | | | | | Population | | | | | | Intervention/Policy | | | | | | Systematic reviews, stakeholders’ documents, and position reviews endorsing respective characteristic |
| --- | --- | --- | --- | --- | --- | --- | --- | --- | --- | --- | --- | --- | --- | --- | --- | --- | --- | --- | --- | --- | --- | --- | --- |
|  | Doc.  type | PA + diet | | | PA | | Diet | Sedentary behavior | | General | | Vulnerable | Child/Adolescents | | Adults | | Intervention and policy | | Intervention | | Policy | |  |
| ***Domain: Reach*** |  |  | | |  | |  |  | | |  |  |  | |  | |  | |  | |  | |  |
| *Strategies facilitating recruitment processes* | | | | | | | | | | | | | | | | | | | | | | | |
| Resources/strategies for implementers helping them to invite and follow-up participants | 1 | | | 1 | | 2 | | |  | |  | 1 | |  | | 1 | 1 | 2 | | 1 | |  | Carroll 2011; Hearn 2008; Heath 2012 |
|  | 3 | | | 1 | |  | | |  | |  |  | |  | |  | 1 | 1 | |  | |  | Besculides 2008 |
| Active recruitment strategies employed | 1 | | | 1 | |  | | |  | |  |  | | 1 | |  |  |  | | 1 | |  | Eakin2002 |
|  | 3 | | | 1 | |  | | |  | |  |  | |  | |  | 1 | 1 | |  | |  | Besculides 2008 |
| Awareness raising (strategies to raise awareness of dietary behavior, physical activity, sedentary behaviors, as well as interventions and policies) to help implementers to invite participants | 1 | | | 1 | | 1 | | | 1 | |  | 2 | | 1 | |  |  | 1 | | 1 | | 1 | Baker 2011; Van De Vijver 2012; Niebylski 2014 |
|  | 2 | | | 3 | |  | | |  | |  | 1 | |  | | 2 |  | 1 | |  | | 2 | Koplan 2005; NICE 2012; Birch 2011 |
|  | 3 | | | 2 | | 1 | | | 4 | |  | 6 | |  | | 1 |  | 1 | | 2 | | 4 | Fransen 2012; Summerbell 2012; Caraher 2007; Craig 2012; Keller 2007;Watts 2011; Capacci 2012 |
| Marketing plans and collaboration with marketing agencies to recruit participants | 1 | | |  | | 1 | | |  | |  | 1 | |  | |  |  | 1 | |  | |  | Baker 2011 |
| Recruitment of organizations which are willing to cooperate | 1 | | | 1 | |  | | |  | |  |  | |  | |  | 1 |  | | 1 | |  | Matthews 2014 |
| Recruitment form community-based settings to increase participation | 1 | | |  | | 1 | | |  | |  |  | |  | |  | 1 | 1 | |  | |  | Wilding 2012 |
| Proactive recruitment to participate in each session | 1 | | | 1 | |  | | |  | |  |  | | 1 | |  |  |  | | 1 | |  | Eakin 2002 |
| Participation mobilization methods, varying across populations | 3 | | | 1 | |  | | |  | |  | 1 | |  | |  |  |  | | 1 | |  | Cambon 2012 |
| Methods of recruitment of those who deliver the program | 1 | | | 1 | |  | | |  | |  |  | |  | |  | 1 |  | | 1 | |  | Laws 2012 |
| Recruitment protocols for external organizations | 1 | | | 1 | |  | | |  | |  |  | |  | |  | 1 |  | | 1 | |  | Matthews 2014 |
| Incentives to participate | 1 | | | 2 | | 1 | | |  | |  |  | |  | |  | 3 |  | | 3 | |  | Vuillemin 2011; Matthews 2014; Wierenga 2013 |
|  | 3 | | | 1 | |  | | |  | |  |  | |  | |  | 1 | 1 | |  | |  | Besculides 2008 |
| Develop a proactive referral system | 3 | | | 1 | |  | | |  | |  |  | |  | |  | 1 | 1 | |  | |  | Besculides 2008 |
| Recruitment form community-based settings to increase participation | 1 | | |  | | 1 | | |  | |  |  | |  | |  | 1 | 1 | |  | |  | Wilding 2012 |
| *Issues in participation processes and their effects on implementation* | | | | | | | | | | | | | | | | | | | | | | |  |
| General attrition rates | 1 | | | 5 | | 3 | | | 1 | | 1 | 1 | |  | | 3 | 6 | 2 | | 8 | |  | Akers 2010; Laws 2012; Matthews 2014; Vuillemin; 2011; Galaviz 2014; Kohl 2013; Klesges 2012; Robertson 2012; Wang 2012; Van Grieken 2012 |
|  | 3 | | | 3 | | 1 | | |  | |  | 3 | | 1 | |  |  | 2 | | 2 | |  | Dzewaltowski 2004; Antikainen 2011; Gillison 2012; Glasgow 2007 |
| Intervention attendance, completion | 1 | | |  | | 3 | | |  | |  | 1 | |  | |  | 2 |  | | 3 | |  | Mcmahon 2012; Galaviz 2014; Robertson 2012 |
| Participation levels i.e., percent of those agreeing among eligible participants | 1 | | | 2 | | 1 | | | 1 | |  | 1 | |  | | 2 | 1 | 4 | |  | |  | Klesges 2012; Hoehner 2013; Lombard 2009; Wang 2012 |
|  | 3 | | | 1 | |  | | |  | |  | 1 | |  | |  |  | 1 | |  | |  | McNeil 2006 |
| Representativeness of attrition and dropout | 1 | | | 4 | | 2 | | |  | |  |  | |  | | 2 | 4 | 2 | | 4 | |  | Klesges 2012; Klesges 2008; Laws 2012; Kohl 2013; Robertson 2012; Vuillemin 2011 |
|  | 3 | | | 1 | |  | | |  | |  | 1 | |  | |  |  | 1 | |  | |  | Glasgow 2007 |
| Differential attrition across the program conditions/types | 1 | | | 3 | | 1 | | |  | |  |  | |  | | 2 | 2 | 2 | | 2 | |  | Klesges 2012; Klesges 2008; Laws 2012; Vuillemin 2011 |
| Participation across the program components (dose received or exposure levels) | 1 | | | 2 | | 1 | | |  | |  |  | |  | | 1 | 2 |  | | 3 | |  | Wierenga 2013; Beets 2009; Kohl 2013 |
| Participant attendance across sessions/session completion rates | 1 | | | 3 | |  | | |  | |  |  | | 1 | |  | 2 |  | | 3 | |  | Akers 2010; Matthews 2014; Eakin 2002 |
| Representativeness of those who adopted the program (staff, settings, or institutions) characteristics of those who adopted vs those who did not) | 1 | | | 2 | |  | | |  | |  | 1 | |  | | 1 |  | 1 | | 1 | |  | Klesges 2008; Rabin 2010 |
|  | 3 | | | 1 | |  | | |  | |  | 1 | |  | |  |  |  | | 1 | |  | Dzewaltowski 2004 |
| Setting and participants representativeness | 1 | | | 1 | |  | | |  | |  | 1 | |  | |  |  |  | | 1 | |  | Goode 2012 |
| Extent to which the target group actually received the intervention | 3 | | | 1 | |  | | |  | |  | 1 | |  | |  |  |  | | 1 | |  | Dzewaltowski 2004 |
| Patient adherence rates | 1 | | |  | | 1 | | |  | |  |  | |  | |  | 1 |  | | 1 | |  | Goodwin 2011 |
| Number and length of components completed | 1 | | | 1 | |  | | |  | |  | 1 | |  | |  |  |  | | 1 | |  | Goode 2012 |
| Provider/implementer representativeness | 3 | | |  | | 1 | | |  | |  | 1 | |  | |  |  |  | | 1 | |  | Eakin 2005 |
| *Cultural and social issues in reaching target populations* | | | | | | | | | | | | | | | | | | | | | | | |
| Enhancing cultural competences of intervention/policy (creating culturally sensitive versions of materials) | 1 | | |  | | 3 | | | 1 | |  |  | | 1 | | 1 | 2 | 3 | |  | | 1 | Sumlin 2012; Carroll 2011; Teufel-Shone 2009; Robertson-Wilson 2012 |
|  | 2 | | | 3 | | 2 | | | 1 | |  | 2 | |  | | 3 | 1 | 1 | |  | | 5 | WHO Europe 2007a  Stallings 2010; Sims 2006; WHO 2008; CDC 2011; WHO 2010 |
|  | 3 | | |  | | 1 | | |  | |  | 1 | |  | |  |  | 1 | |  | |  | Kahn 2002 |
| Addressing the place, race, occupation, gender, religion, education, SES, social status | 1 | | | 2 | |  | | |  | |  |  | | 1 | | 1 |  | 1 | | 1 | |  | Waters 2011; Nierkens 2013 |
|  | 2 | | |  | | 1 | | |  | |  | 1 | |  | |  |  |  | |  | | 1 | WHO 2010 |
| Barriers for implementation vary across more and less disadvantaged populations | 1 | | | 1 | |  | | |  | |  |  | | 1 | |  |  |  | | 1 | |  | Eakin 2002 |
| ***Domain: Efficacy*** | | | | | | | | | | | | | | | | | | | | | | | |
| *Satisfaction with implementation* | | | | | | | | | | | | | | | | | | | | | | | |
| Participants’ satisfaction with implementation | 1 | | | 3 | |  | | | 1 | |  |  | |  | | 2 | 2 | 2 | | 2 | |  | Wierenga 2013; Matthews 2014; Waters 2011; Wang 2012 |
| Staff satisfaction with implementation | 1 | | | 2 | |  | | |  | |  |  | |  | | 1 | 1 | 1 | | 1 | |  | Matthews 2014; Waters 2011 |
| *Feasibility and acceptability* | | | | | | | | | | | | | | | | | | | | | | | |
| Level of feasibility of implementation perceived by participants | 1 | | | 2 | |  | | |  | |  | 1 | |  | | 1 |  | 1 | | 1 | |  | Goode 2012; Waters 2011 |
|  | 3 | | | 1 | |  | | |  | |  | 1 | |  | |  |  |  | | 1 | |  | Cambon 2012 |
| Feasibility of implementation and acceptability of implementation among providers, stakeholders, and participants | 1 | | | 6 | | 1 | | |  | |  | 2 | |  | | 4 | 1 | 3 | | 4 | |  | Flynn 2006; Leung 2012; Brennan 2014; Hoehner 2013; Rabin 2010; Langford 2014; Laws 2012 |
|  | 3 | | | 4 | |  | | |  | |  | 3 | |  | | 1 |  | 4 | |  | |  | Khan 2009; Glasgow 2007; Gillison 2012; McNeil 2006 |
| Acceptability of the program among participants (e.g., acceptability of: the group size, the type of participants, interventionists’ skills) | 1 | | | 2 | | 2 | | |  | |  | 2 | |  | | 1 | 1 | 2 | | 1 | | 1 | McMahon 2012; Hoehner 2013; Waters 2011;  Diepeveen 2013 |
|  | 3 | | | 2 | |  | | | 1 | |  | 2 | |  | | 1 |  |  | | 2 | | 1 | Cambon 2012; Summerbell 2012; Caraher 2007 |
| Level of feasibility perceived by staff | 1 | | | 1 | | 1 | | |  | |  |  | |  | | 1 | 1 | 1 | | 1 | |  | Goodwin 2011; Waters 2011 |
| Importance and practicality of guideline | 1 | | |  | | 1 | | |  | |  |  | |  | |  | 1 |  | | 1 | |  | Goodwin 2011 |
| Feasibility of all components delivered | 1 | | |  | | 1 | | |  | |  | 1 | |  | |  |  |  | | 1 | |  | Blackman 2013 |
| Feasibility of implementation in routine clinical care settings | 1 | | | 1 | |  | | |  | |  |  | |  | |  | 1 |  | | 1 | |  | Cardona-Morrell 2010 |
| Ethical and legal feasibility evaluated | 1 | | | 1 | |  | | |  | |  |  | |  | | 1 |  | 1 | |  | |  | Flynn 2006 |
| Acceptability among providers | 3 | | | 2 | |  | | |  | |  | 1 | |  | | 1 |  | 1 | | 1 | |  | Summerbell 2012; Gillison 2012 |
| Acceptability among policy makers | 1 | | | 1 | |  | | |  | |  | 1 | |  | |  |  |  | |  | | 1 | Diepeveen 2013 |
| *Evaluation of implementation/adoption processes (excluding evaluation of the outcomes of the program)* | | | | | | | | | | | | | | | | | | | | | | | |
| Benefits of the intervention to be observable by those intending to adopt the intervention | 1 | | | 1 | | 1 | | |  | |  |  | |  | |  | 2 |  | | 2 | |  | Goodwin 2011; Wierenga 2013 |
|  | 3 | | | 1 | |  | | |  | |  | 1 | |  | |  |  | 1 | |  | |  | Gillison 2012 |
| Benefits observed by target population (relative advantage, compared to pre-intervention) | 1 | | | 1 | |  | | |  | |  |  | |  | |  | 1 |  | | 1 | |  | Wierenga 2013 |
|  | 3 | | | 2 | |  | | |  | |  | 2 | |  | |  |  | 1 | | 1 | |  | Gillison 2012; Cambon 2012 |
| Evaluation and monitoring results are disseminated to communities, stakeholders, and nationally | 2 | | | 4 | | 1 | | |  | |  | 4 | |  | | 1 |  | 1 | |  | | 4 | WHO Europe 2007a;  NICE 2012; WHO 2008; WHO 2010; Glickman 2012 |
| Difficulty/a lack of opportunity to assess the impact of one policy separately from ancillary policies/interventions due to the increasing complexity of policies/legislations | 1 | | |  | | 1 | | | 1 | |  | 1 | |  | | 1 |  |  | |  | | 2 | Niebylski 2014; Robertson-Wilson 2012 |
|  | 3 | | | 2 | |  | | |  | |  |  | | 1 | | 1 |  |  | |  | | 2 | Gaziano 2007; Ramanathan 2008 |
| Program results evaluated as useful by stakeholders, interventionists, funding agencies | 1 | | | 1 | |  | | |  | |  |  | |  | | 1 |  | 1 | |  | |  | Flynn 2006 |
| Organization and managers observe benefits of the program | 1 | | | 1 | |  | | |  | |  |  | |  | |  | 1 |  | | 1 | |  | Wierenga 2013 |
|  | 3 | | | 1 | |  | | |  | |  | 1 | |  | |  |  | 1 | |  | |  | Gillison 2012 |
| ‘Successful implementation’ defined and measured (effects’ indicators: targets measurable and their timeframe specified) | 3 | | |  | | 1 | | |  | |  | 1 | |  | |  |  |  | |  | | 1 | Bellew 2008 |
| ***Domain: Adoption*** | | | | | | | | | | | | | | | | | | | | | | | |
| *Training for implementation* | | | | | | | | | | | | | | | | | | | | | | | |
| Training for implementers and disseminators (e.g., training, certifıcates, workshops, training instructions, skill development) | 1 | | | 11 | | 7 | | | 2 | |  | 5 | | 5 | | 4 | 6 | 7 | | 13 | |  | Rabin 2010; Goodwin 2011; Mcmahon 2012; Goode 2012; Matthews 2014; Vuillemin 2014; Hoehner 2013; Waters 2011; Hearn 2008; Sahay 2006; Carroll 2011; Heath 2012; Laws 2012; Teufel-Shone 2009; Van De Vijver 2012; Nierkens 2013; Wang 2012; Verstraeten 2012; Langford 2014; Fleury 2009 |
|  | 2 | | | 3 | | 1 | | | 2 | |  | 2 | |  | | 3 | 1 | 1 | | 1 | | 4 | EC 2006; NICE 2009; NICE 2012; Birch 2011; Stallings 2010; WHO 2013 |
|  | 3 | | | 5 | | 4 | | | 2 | |  | 5 | | 1 | | 4 | 1 | 5 | | 4 | | 2 | ADA 2003; Stockley 2001; Cambon 2012; Glasgow 2007; Kahn 2002; Besculides 2008; Antikainen 2011; Ribeiro 2010; Taylor 2013; Keller 2007; Tremblay 2012 |
| Training for staff in aspects of implementation and facilitation of inter-sectorial collaboration | 1 | | | 1 | | 1 | | |  | |  | 1 | |  | | 1 |  | 1 | | 1 | |  | Heath 2012; Klesges 2012 |
|  | 3 | | | 1 | |  | | |  | |  | 1 | |  | |  |  | 1 | |  | |  | Glasgow 2007 |
| Training instructions/materials for implementers | 1 | | | 2 | |  | | | 1 | |  | 1 | |  | | 1 | 1 | 1 | | 2 | |  | Rabin 2010; Matthews 2014; Wang 2012 |
|  | 3 | | | 2 | |  | | | 1 | |  | 2 | |  | | 1 |  |  | | 2 | | 1 | Cambon 2012; Taylor 2013; Keller 2007 |
| Technical assistance for dissemination and implementation approaches | 1 | | | 1 | |  | | |  | |  | 1 | |  | |  |  |  | | 1 | |  | Rabin 2010 |
|  | 2 | | | 1 | |  | | | 1 | |  | 1 | |  | | 1 |  | 1 | |  | | 1 | WHO Europe 2007a; Stallings 2010 |
| Regular meetings or supervision for staff to secure implementation | 1 | | | 2 | |  | | |  | |  |  | | 1 | |  | 1 |  | | 2 | |  | Matthews 2014; Verstraeten 2012 |
|  | 3 | | | 2 | | 1 | | |  | |  |  | | 1 | | 1 | 1 | 1 | | 2 | |  | Antikainen 2011; Besculides 2008; Taylor 2013 |
| Developing clinical goals for implementers | 1 | | | 1 | |  | | |  | |  |  | |  | | 1 |  | 1 | |  | |  | Hearn 2008 |
|  | 3 | | |  | | 1 | | |  | |  |  | | 1 | |  |  |  | | 1 | |  | Antikainen 2011 |
| Education and training for the ‘social environment’ of implementation (local public health practitioners, community health workers and researchers) | 1 | | |  | | 1 | | |  | |  |  | | 1 | |  |  | 1 | |  | |  | Teufel-Shone 2009 |
| Multimodal training in dissemination and implementation strategies | 1 | | | 1 | |  | | |  | |  | 1 | |  | |  |  |  | | 1 | |  | Rabin 2010 |
| Evaluation/feedback for staff involved | 1 | | | 1 | |  | | |  | |  |  | |  | |  | 1 |  | | 1 | |  | Matthews 2014 |
|  | 2 | | |  | | 1 | | |  | |  |  | |  | | 1 |  | 1 | |  | |  | NICE 2009 |
| Documentation of training (e.g., for LHA roles varied from 4 to 45 h, and included educational seminars, role playing, and interactive practice sessions targeting education, support, and behavioral change) | 1 | | | 1 | |  | | |  | |  |  | | 1 | |  |  |  | | 1 | |  | Fleury 2009 |
| *Staff expertise for implementation* | | | | | | | | | | | | | | | | | | | | | | | |
| No additional expertise required for staff involved in implementation | 1 | | | 1 | | 1 | | |  | |  |  | |  | | 1 | 1 | 1 | | 1 | |  | Vuillemin 2011; Klesges 2008 |
|  | 3 | | | 1 | | 1 | | |  | |  | 1 | |  | |  | 1 | 1 | | 1 | |  | Gillison 2012; White 2009 |
| Implementers’ skill, knowledge, and competence to implement the program correctly | 1 | | | 3 | | 3 | | |  | |  | 1 | | 1 | | 2 | 2 | 1 | | 4 | |  | Wierenga 2013; Huijg 2014; Hearn 2008; Beets 2009; Carroll 2011; Nierkens 2013 |
|  | 2 | | |  | | 1 | | |  | |  |  | |  | | 1 |  | 1 | |  | |  | NICE 2009 |
|  | 3 | | | 1 | |  | | | 1 | |  | 2 | |  | |  |  | 1 | |  | | 1 | Keller 2007; Glasgow 2007 |
| Communication/collaboration between professionals implementing the program | 1 | | |  | | 1 | | |  | |  |  | |  | |  | 1 |  | | 1 | |  | Goodwin 2011 |
|  | 2 | | | 1 | |  | | |  | |  | 1 | |  | |  |  |  | |  | | 1 | NICE 2012 |
| *Collaboration and communication for implementation* | | | | | | | | | | | | | | | | | | | | | | | |
| Collaboration between implementers; the use of methods to increase communication between implementers | 1 | | | 2 | | 1 | | |  | |  |  | |  | | 1 | 2 | 2 | | 1 | |  | Wierenga 2012; Hearn 2008; Carroll 2011 |
|  | 2 | | | 3 | | 1 | | |  | |  | 2 | |  | | 1 | 1 |  | | 1 | | 3 | NICE 2012; CDC 2011; WHO 2010; EC 2006 |
|  | 3 | | | 2 | |  | | | 1 | |  | 2 | |  | |  | 1 | 3 | |  | |  | Besculides 2008; King 2011; Stockley 2001 |
| Relationship and communication channels between policy-makers and implementers for successful implementation | 2 | | | 1 | |  | | |  | |  | 1 | |  | |  |  | 1 | |  | |  | WHO Europe 2007a |
| Key political and stakeholders’ support for implementation (stakeholders identified and involved) | 1 | | |  | | 1 | | | 1 | |  |  | |  | |  | 2 | 2 | |  | |  | Carroll 2011; Geaney 2013 |
|  | 2 | | | 1 | | 1 | | | 1 | |  | 2 | |  | | 1 |  |  | |  | | 3 | WHO 2007; CDC 2011; WHO 2013 |
|  | 3 | | |  | | 2 | | | 2 | |  | 3 | |  | | 1 |  | 1 | |  | | 3 | Bellew 2008; Thow 2011; Tremblay 2012; Stockley 2001 |
| Cross-sectorial collaboration: collaboration between sectors of health, sports, food, transportation, planning and housing, green spaces, education, healthcare, and social services | 2 | | | 3 | | 3 | | | 2 | |  | 4 | |  | | 3 | 1 | 3 | |  | | 5 | Koplan 2005; WHO Europe 2007a; Sims 2006; WHO 2007; Glickman 2012; Stallings 2010; WHO 2013; NICE 2009 |
|  | 3 | | | 3 | | 5 | | | 3 | |  | 7 | |  | | 4 |  | 3 | | 1 | | 7 | ADA 2003; Fransen 2012; Ribeiro 2010; Kahn 2002; Bundy 2012; Crammond 2013;  Shill 2012b Thow 2011; Christiansen 2012; Craig 2012; Freudenberg 2010 |
| Involvement of multiple stakeholders at multiple levels | 2 | | | 1 | | 2 | | | 2 | |  | 3 | |  | | 2 |  | 1 | |  | | 4 | Stallings 2010; WHO 2007; NICE 2009; NICE 2012; WHO 2013 |
|  | 3 | | |  | | 1 | | | 1 | |  | 2 | |  | |  |  |  | |  | | 2 | Christiansen 2012; Keller 2007 |
| Collaboration with professionals and organizations for program implementation | 1 | | |  | | 3 | | |  | |  | 3 | |  | |  |  | 1 | | 2 | |  | Huijg 2014; Heath 2006; Heath 2012 |
|  | 3 | | | 2 | |  | | |  | |  | 1 | |  | | 1 |  | 2 | |  | |  | Fransen 2012; Pratt 2008 |
| Collaboration between professional staff and volunteers | 2 | | | 1 | |  | | |  | |  |  | |  | |  | 1 |  | | 1 | |  | EC 2006 |
| Leadership supporting implementation at target setting (e.g., schools, organizations) | 2 | | | 1 | | 1 | | |  | |  | 1 | |  | |  | 1 | 1 | |  | | 1 | NICE 2008; NICE 2012 |
|  | 3 | | |  | |  | | | 1 | |  |  | |  | | 1 |  |  | |  | | 1 | Carlsson 2008 |
| Effective leadership to secure collaboration (between facilitators, institutions, and organizations involved) | 1 | | | 1 | | 1 | | |  | |  |  | |  | |  | 2 |  | | 2 | |  | Goodwin 2011; Wierenga 2013 |
|  | 2 | | | 5 | | 2 | | | 2 | |  | 4 | |  | | 5 |  | 1 | |  | | 8 | Stallings 2010; WHO 2007; WHO 2008; CDC 2011; Birch 2011; Glickman 2012; NICE 2012; NICE 2009; WHO 2013 |
| Leadership: appointment of a national coordinator | 2 | | |  | |  | | | 1 | |  | 1 | |  | |  |  |  | |  | | 1 | WHO 2013 |
| Synergy with other existing or operating programs | 3 | | | 2 | | 2 | | | 4 | |  | 6 | |  | | 1 | 1 | 1 | | 1 | | 6 | Fransen 2012; Sorensen 2004; Craig 2012; Crammond 2013, Freudenberg 2010; Perez-Ferrerr 2010; Shill 2012b; Thow 2011 |
| Securing food industry involvement/preventing and counteracting food industry resistance | 2 | | |  | |  | | | 1 | |  |  | |  | | 1 |  |  | |  | | 1 | Stallings 2010 |
|  | 3 | | | 1 | |  | | | 4 | |  | 4 | |  | | 1 |  |  | | 1 | | 4 | Freudenberg 2010; Keller 2007; Perez-Ferrer 2010; Shill 2012a; Watts 2011 |
| Stakeholders input at every step in program development, implementation and evaluation | 1 | | | 1 | |  | | |  | |  |  | |  | | 1 |  | 1 | |  | |  | Flynn 2006 |
|  | 3 | | | 1 | | 1 | | |  | |  | 2 | |  | |  |  | 1 | | 1 | |  | McNeil 2006; Eakin 2005 |
| Stakeholders involved in planning implementation | 3 | | |  | |  | | | 1 | |  | 1 | |  | |  |  | 1 | |  | |  | Stockley 2001 |
| Stakeholders acceptance for the program | 1 | | | 1 | |  | | |  | |  |  | |  | |  | 1 |  | | 1 | |  | Laws 2012 |
| Stakeholders awareness about an issue (e.g., trans fatty acids as a problem) | 3 | | |  | |  | | | 1 | |  | 1 | |  | |  |  |  | |  | | 1 | Perez-Ferrer 2010 |
| Addressing challenges and needs of stakeholders | 3 | | | 1 | |  | | |  | |  |  | |  | | 1 |  | 1 | |  | |  | Trudnak 2012 |
| Incentives for the beneficiaries/organizations to facilitate and support their participation | 3 | | | 1 | |  | | |  | |  | 1 | |  | |  |  |  | | 1 | |  | Cambon 2012 |
| An organized consumer lobby to influence government/industry | 3 | | |  | |  | | | 1 | |  | 1 | |  | |  |  |  | |  | | 1 | Perez-Ferrer 2010 |
| Voluntary agreements with food industry promoting behavior change (e.g., to reduce trans fatty acids) | 3 | | |  | |  | | | 1 | |  | 1 | |  | |  |  |  | |  | | 1 | Perez-Ferrer 2010 |
| Involving local business | 2 | | | 1 | |  | | |  | |  | 1 | |  | |  |  |  | |  | | 1 | NICE 2012 |
| Competitive business environment | 1 | | | 1 | |  | | |  | |  |  | |  | |  | 1 |  | | 1 | |  | Wierenga 2013 |
| Support from agencies outside of the institution where the program is implemented (statutory and voluntary sectors) | 2 | | |  | | 1 | | |  | |  |  | |  | |  | 1 | 1 | |  | |  | NICE 2008 |
| Presence of advisory board: well-functioning advisory board | 1 | | | 1 | |  | | |  | |  |  | |  | |  | 1 |  | | 1 | |  | Wierenga 2012 |
| Establish coordinating team which includes members from all stakeholder groups (promote integration of all stakeholders) | 2 | | | 1 | |  | | |  | |  |  | |  | | 1 |  |  | |  | | 1 | WHO 2008 |
| Conflicting interests and methods of dealing with conflicts (e.g., within/between agency implementing policy/intervention) | 1 | | | 1 | |  | | |  | |  |  | |  | |  | 1 |  | | 1 | |  | Wierenga 2012 |
| Developing an explicit framework (linking the main actors, policy tools and settings) | 2 | | | 1 | |  | | |  | |  | 1 | |  | |  |  | 1 | |  | |  | WHO Europe 2007a |
| *Community support for implementation* | | | | | | | | | | | | | | | | | | | | | | | |
| Securing the involvement of local community in implementation | 1 | | | 1 | |  | | | 2 | |  | 1 | |  | | 2 |  | 2 | |  | | 1 | Wang 2012; Niebylski 2014; Waters 2011 |
|  | 2 | | | 2 | | 2 | | | 1 | |  | 4 | |  | | 1 |  | 1 | |  | | 4 | NICE 2012; WHO Europe 2007b; WHO 2007; Koplan 2005; WHO 2013 |
|  | 3 | | |  | | 1 | | | 1 | |  | 2 | |  | |  |  |  | |  | | 2 | Christiansen 2012; Caraher 2007 |
| Community organizations support adoption | 1 | | | 2 | |  | | |  | |  |  | | 1 | |  | 1 |  | | 2 | |  | Matthews 2014; Fleury 2009 |
|  | 2 | | | 3 | | 2 | | |  | |  | 4 | |  | | 1 |  | 2 | |  | | 3 | NICE 2012; WHO Europe 2007a; WHO 2007; Koplan 2005; WHO Europe 2007b |
|  | 3 | | | 2 | |  | | |  | |  | 1 | |  | |  | 1 | 1 | |  | |  | Besculides 2008; King 2011 |
| Building relationships/networks for implementation (between implementing organizations and community organizations) | 1 | | |  | | 2 | | |  | |  | 1 | |  | |  | 1 | 1 | | 1 | |  | Huijg 2014; Carroll 2011 |
|  | 2 | | | 1 | |  | | |  | |  | 1 | |  | |  |  |  | |  | | 1 | NICE 2012 |
|  | 3 | | |  | |  | | | 1 | |  |  | |  | | 1 |  |  | |  | | 1 | Carlsson 2008 |
| Implementation integrated into existing local community programs/guidelines | 1 | | |  | | 1 | | |  | |  | 1 | |  | |  |  | 1 | |  | |  | Hoehner 2013 |
|  | 3 | | |  | |  | | | 1 | |  | 1 | |  | |  |  |  | |  | | 1 | Keller 2007 |
| Local community leadership involved in implementation and maintenance | 1 | | |  | | 1 | | |  | |  |  | | 1 | |  |  | 1 | |  | |  | Teufel-Shone 2009 |
|  | 2 | | | 1 | | 1 | | |  | |  | 1 | |  | | 1 |  | 1 | |  | | 1 | NICE 2009; NICE 2012 |
| Community analysis to plan implementation | 1 | | |  | | 1 | | |  | |  | 1 | |  | |  |  |  | | 1 | |  | Heath 2012 |
|  | 3 | | | 1 | |  | | |  | |  | 1 | |  | |  |  | 1 | |  | |  | King 2011 |
| Developing community goals referring to behavior change | 1 | | | 1 | |  | | |  | |  |  | |  | | 1 |  | 1 | |  | |  | Hearn 2008 |
| Community needs identified and accounted for in implementation | 1 | | | 2 | |  | | |  | |  |  | | 2 | |  |  |  | | 2 | |  | Fleury 2009; Van De Vijver 2012 |
| Community associations receive training facilitating the use of policy | 3 | | |  | |  | | | 1 | |  | 1 | |  | |  |  |  | |  | | 1 | Keller 2007 |
| The use of peer or lay volunteer to implement program and support maintenance | 2 | | | 1 | | 1 | | |  | |  |  | |  | |  | 2 |  | | 2 | |  | Goodwin 2011; Matthews 2014 |
| Integration: divergent interest in community, funding agents, those implementing programs accounted for | 3 | | |  | |  | | | 1 | |  | 1 | |  | |  |  |  | |  | | 1 | Caraher 2007 |
| Materials integration: materials employed use guidelines which are already familiar to communities, specific target groups (e.g., children) | 3 | | |  | |  | | | 1 | |  | 1 | |  | |  |  |  | |  | | 1 | Keller 2007 |
| Materials applicable by community agencies | 3 | | |  | | 1 | | |  | |  |  | |  | |  | 1 |  | | 1 | |  | White 2009 |
| Integration with existing community infrastructures | 3 | | | 2 | |  | | |  | |  | 1 | |  | | 1 |  | 2 | |  | |  | King 2011; Pratt 2008 |
| Community social environment barriers to implementation (e.g., safety concerns, transportation costs in the community) accounted for | 2 | | | 1 | | 1 | | |  | |  |  | |  | | 1 | 1 | 1 | |  | | 1 | Sims 2006; Koplan 2005 |
| *Adoption in physical environment facilitating implementation* | | | | | | | | | | | | | | | | | | | | | | | |
| Maintenance or development of built and natural environment to enable policies implementation | 1 | | |  | | 2 | | | 1 | |  | 1 | | 1 | |  | 1 | 3 | |  | |  | Teufel-Shone 2009; Baker 2011; Geaney 2013 |
|  | 2 | | | 3 | | 3 | | |  | |  | 2 | |  | | 3 | 1 | 1 | |  | | 5 | Birch 2011; Sims 2006; WHO Europe 2007b; NICE 2009; CDC 2011; Glickman 2012 |
|  | 3 | | | 1 | | 2 | | |  | |  | 2 | |  | | 1 |  |  | |  | | 3 | Christiansen 2012; Ramanathan 2008; Shill 2012b |
| Supportive physical environment in the community promotes implementation and adoption | 1 | | | 1 | |  | | |  | |  |  | |  | | 1 |  |  | | 1 | |  | Langford 2014 |
|  | 2 | | | 2 | | 2 | | |  | |  | 1 | |  | | 2 | 1 | 1 | |  | | 3 | Birch 2011, Sims 2006; WHO Europe 2007b; Koplan 2005 |
|  | 3 | | |  | | 1 | | |  | |  | 1 | |  | |  |  |  | |  | | 1 | Craig 2011 |
| Development physical environment for people with disabilities | 2 | | | 1 | |  | | |  | |  |  | |  | | 1 |  |  | |  | | 1 | Birch 2011 |
| Environmental barriers to implementation (infrastructure, transport, urban design) accounted for | 2 | | | 1 | | 1 | | |  | |  | 1 | |  | | 1 |  | 1 | |  | | 1 | WHO Europe 2007b; Koplan 2005 |
| Integration with environmental changes | 3 | | | 1 | |  | | |  | |  |  | |  | | 1 |  | 1 | |  | |  | Hoelscher 2013 |
| *Governmental and legislative involvement* | | | | | | | | | | | | | | | | | | | | | | | |
| Federal (national) government co-issues the program or is involved in program issuing | 1 | | |  | |  | | | 1 | |  | 1 | |  | |  |  |  | |  | | 1 | Niebylski 2014 |
|  | 2 | | |  | | 2 | | | 1 | |  | 3 | |  | |  |  |  | |  | | 3 | WHO Europe 2007b; WHO 2007; WHO 2013 |
|  | 3 | | | 2 | | 1 | | | 2 | |  | 3 | |  | | 2 |  | 1 | |  | | 4 | Christiansen 2012; Freudenberg 2010; Shill 2012a; Carlsson 2008; Fransen 2012 |
| Securing political commitment to the program (e.g., by specific implementation plans) | 1 | | | 1 | |  | | |  | |  | 1 | |  | |  |  |  | |  | | 1 | Diepeveen 2013 |
|  | 3 | | |  | | 1 | | | 1 | |  | 2 | |  | |  |  |  | |  | | 2 | Christiansen 2012; Thow 2011 |
| Legal basis/secured legal support for implementation and maintenance (e.g. fiscal, liability instruments, market environment laws) | 2 | | | 1 | |  | | | 1 | |  | 1 | |  | | 1 |  |  | |  | | 2 | WHO 2008; WHO 2013 |
|  | 3 | | |  | |  | | | 2 | |  | 1 | |  | | 1 |  |  | |  | | 2 | Bundy 2012; Capacci 2012 |
| Collaboration and division of labor between national government structures (e.g., ministries) | 2 | | |  | | 1 | | |  | |  | 1 | |  | |  |  |  | |  | | 1 | WHO Europe 2007b |
| Accounting for legal instruments to support implementation (existing legal instruments supporting implementation, changes in law, and legal burden for businesses) | 3 | | |  | |  | | | 4 | |  | 4 | |  | |  |  |  | |  | | 4 | Capacci 2012; Caraher 2007; Crammond 2013; Thow 2011 |
| Politicians’ collaboration (negotiation with and influencing politicians and policy makers) | 3 | | | 2 | |  | | | 2 | |  | 1 | |  | | 3 |  |  | |  | | 4 | Carlsson 2008; Freudenberg 2010; Crammond 2013; Ramanathan 2008 |
| Involvement of a local government and accounting for regional regulations | 2 | | | 1 | | 2 | | |  | |  | 1 | |  | | 1 | 1 | 1 | |  | | 2 | WHO 2007; Sims 2006; Koplan 2005 |
|  | 3 | | | 2 | | 1 | | | 1 | |  | 2 | |  | | 2 |  | 1 | |  | | 3 | Larson 2012; Freudenberg 2010; Caraher 2007; Craig 2012 |
| Accounting for conflicting policies in adoption process | 3 | | |  | | 2 | | | 2 | |  | 4 | |  | |  |  |  | |  | | 4 | Shill 2012a; Shill 2012b; Rutten 2013; Keller 2007 |
| Collaboration between municipal, state and federal (national) government in implementation (or at least acceptance at higher levels) | 3 | | | 1 | |  | | |  | |  |  | |  | | 1 |  |  | |  | | 1 | Freudenberg 2010 |
|  | 2 | | |  | | 1 | | |  | |  | 1 | |  | |  |  |  | |  | | 1 | WHO Europe 2007b |
| Avoiding competition between levels of government for power | 3 | | | 1 | |  | | |  | |  |  | |  | | 1 |  |  | |  | | 1 | Freudenberg 2010 |
| Communication within governmental structures about a policy | 3 | | |  | |  | | | 1 | |  | 1 | |  | |  |  |  | |  | | 1 | Keller 2007 |
| Government resistance to issue specific policies (e.g., trans fatty acids) | 3 | | |  | |  | | | 1 | |  | 1 | |  | |  |  |  | |  | | 1 | Perez-Ferrer 2012 |
| Advocacy - interest groups working to further certain goals (making demands to government) | 3 | | |  | | 1 | | |  | |  | 1 | |  | |  |  |  | |  | | 1 | Craig 2012 |
| Securing transition from local/small scale initiatives to nationally owned (governmental) | 3 | | |  | |  | | | 1 | |  |  | |  | | 1 |  |  | |  | | 1 | Bundy 2012 |
| Indirect support of implementation: other government actions/ legal instruments (e.g., to encourage private-sector actions supporting implementation) | 3 | | |  | |  | | | 1 | |  | 1 | |  | |  |  |  | |  | | 1 | Capacci 2012 |
| Constantly changing legal (policy) context results in difficulties to plan for future funds | 3 | | |  | |  | | | 1 | |  | 1 | |  | |  |  |  | |  | | 1 | Caraher 2007 |
| Law reform unlikely if proposed policies are complex | 3 | | |  | |  | | | 1 | |  | 1 | |  | |  |  |  | |  | | 1 | Crammond 2013 |
| Legal regulations placing large/undue burden upon business are barriers to policy implementation | 3 | | |  | |  | | | 1 | |  | 1 | |  | |  |  |  | |  | | 1 | Crammond 2013 |
| Building long-term political support for programs (e.g., on based on collaborative research providing evidence) | 1 | | |  | | 1 | | |  | |  | 1 | |  | |  |  |  | | 1 | |  | Huijg 2014 |
|  | 3 | | |  | |  | | | 1 | |  |  | |  | | 1 |  |  | |  | | 1 | Carlsson 2008 |
| Negotiating with politicians - political processes - in forming policy proposals | 3 | | | 1 | |  | | |  | |  |  | |  | | 1 |  |  | |  | | 1 | Freudenberg 2010 |
| Politicians as executive decision makers - barriers to implementation | 3 | | |  | |  | | | 1 | |  | 1 | |  | |  |  |  | |  | | 1 | Crammond 2013 |
| Policy-makers collaboration with researchers | 3 | | | 1 | |  | | |  | |  |  | |  | | 1 |  |  | |  | | 1 | Ramanathan 2008 |
| Municipal government involved | 3 | | | 1 | |  | | |  | |  |  | |  | | 1 |  |  | |  | | 1 | Freudenberg 2010 |
| Large municipalities - implementation responsibilities assigned to boroughs/districts as a strategy to enable local authorities | 3 | | | 1 | |  | | |  | |  |  | |  | | 1 |  |  | |  | | 1 | Freudenberg 2010 |
| A lack of provincial regulations/policies | 3 | | |  | |  | | | 1 | |  | 1 | |  | |  |  |  | |  | | 1 | Caraher 2007 |
| Lack of political support | 2 | | | 1 | |  | | |  | |  |  | |  | |  | 1 |  | | 1 | |  | EC 2006 |
| Existence of state/regional government regulations for specific settings (e.g., schools, child-care settings) regarding diet/physical activity exist | 3 | | | 1 | |  | | |  | |  |  | |  | | 1 |  | 1 | |  | |  | Larson 2012 |
| Political prioritization (local and state level) | 2 | | | 1 | | 1 | | |  | |  | 1 | |  | | 1 |  | 1 | |  | | 1 | WHO 2007; Koplan 2005 |
| Implementation executed by this level of local authority which has actual responsibility for health care/public health | 3 | | | 1 | |  | | |  | |  |  | |  | | 1 |  |  | |  | | 1 | Freudenberg 2010 |
| Involving provincial coalitions and departments of health, sport, and education | 3 | | |  | | 1 | | |  | |  | 1 | |  | |  |  |  | |  | | 1 | Craig 2012 |
| Local government involved in inter-sectoral collaboration | 2 | | |  | | 1 | | |  | |  |  | |  | |  | 1 |  | |  | | 1 | Sims 2006 |
| Interdependencies (degree to which policy problems can be solved within one policy domain or by one government agency) | 3 | | |  | | 1 | | | 1 | |  | 2 | |  | |  |  |  | |  | | 2 | Rutten 2013; Shill 2012a |
| Existing agricultural policies may be a barrier for food policies | 3 | | |  | |  | | | 1 | |  | 1 | |  | |  |  |  | |  | | 1 | Keller 2007 |
| Conflicting/competing policies or agendas exist | 3 | | |  | | 1 | | | 1 | |  | 2 | |  | |  |  |  | |  | | 2 | Shill 2012a; Shill 2012b |
| ***Domain: consistency, cost, and adaptations in Implementation*** | | | | | | | | | | | | | | | | | | | | | | | |
| *Simplicity as factor facilitating implementation* | | | | | | | | | | | | | | | | | | | | | | | |
| Simplicity of communicating and implementing the program (not too complex, not too difficult to follow) | 1 | | | 3 | | 1 | | |  | | 1 |  | |  | | 3 | 2 | 2 | | 3 | |  | Goodwin 2011; Wierenga 2012; Brennan 2014; Waters 2011; Van Grieken 2012 |
|  | 2 | | |  | | 1 | | |  | |  | 1 | |  | |  |  |  | |  | | 1 | WHO 2010 |
|  | 3 | | | 1 | | 1 | | | 1 | |  |  | |  | | 1 | 2 |  | | 3 | |  | Summerbell 2012; White 2009; Sorensen 2004 |
| Complexities of existing policies and their interrelations as barriers to implementation | 1 | | |  | | 1 | | |  | |  |  | |  | | 1 |  |  | |  | | 1 | Robertson-Wilson 2012 |
|  | 3 | | | 2 | |  | | | 1 | |  | 1 | | 1 | | 1 |  |  | |  | | 3 | Gaziano 2007; Ramanathan 2008; Crammond 2013 |
| Complexity of/diversity of policies and practices targeting physical activity (also, coexisting policies discouraging physical activity) | 1 | | |  | | 1 | | |  | |  |  | |  | | 1 |  |  | |  | | 1 | Robertson-Wilson 2012 |
| *Accessibility* *for participants* | | | | | | | | | | | | | | | | | | | | | | | |
| Increasing accessibility to environmental structures | 2 | | | 2 | | 1 | | |  | |  | 1 | |  | | 1 | 1 | 1 | |  | | 2 | Sims 2006; Glickman 2012; Koplan 2005 |
|  | 3 | | |  | | 1 | | |  | |  | 1 | |  | |  |  |  | |  | | 1 | Christiansen 2012 |
| Financially accessible programs (low-cost, high affordability) | 1 | | | 1 | |  | | |  | |  |  | |  | |  | 1 |  | | 1 | |  | Wierenga 2013 |
|  | 2 | | | 1 | |  | | |  | |  |  | |  | | 1 |  | 1 | |  | |  | Koplan 2005 |
|  | 3 | | | 3 | | 1 | | | 2 | |  | 4 | |  | | 1 | 1 | 3 | |  | | 3 | Pratt 2008; Christiansen 2012; Caraher 2007; Keller 2007; Besculides 2008; Gillison 2012 |
| Barriers for accessibility in physical environment (e.g., architectural solutions as barriers to exercise; a lack of stairs) | 1 | | |  | | 1 | | |  | |  |  | |  | |  | 1 |  | | 1 | |  | Benjamin 2014 |
|  | 2 | | | 1 | | 1 | | |  | |  |  | |  | | 1 | 1 | 1 | |  | | 1 | Sims 2006; Koplan 2005 |
|  | 3 | | |  | | 2 | | |  | |  | 1 | |  | |  | 1 | 1 | | 1 | |  | Kahn 2002; Soler 2010 |
| Accessibility of materials to participants | 3 | | | 1 | | 1 | | |  | |  | 1 | |  | | 1 |  | 1 | | 1 | |  | Summerbell 2014; Kahn 2002 |
| Strategies to increase accessibility to intervention | 1 | | | 1 | |  | | |  | |  |  | |  | | 1 |  | 1 | |  | |  | Ayliffe 2012 |
|  | 3 | | | 1 | |  | | |  | |  |  | |  | | 1 |  |  | |  | | 1 | Freudenberg 2010 |
| Accessibility to practitioners (far location of practitioners) | 3 | | |  | |  | | | 1 | |  |  | |  | |  | 1 | 1 | |  | |  | Haughton 2012 |
| Low accessibility to existing facilities | 2 | | | 1 | |  | | |  | |  |  | |  | | 1 |  | 1 | |  | |  | Koplan 2005 |
|  | 3 | | |  | | 1 | | | 1 | |  | 2 | |  | |  |  |  | |  | | 2 | Caraher 2007; Christiansen 2012 |
| Availability of healthy foods (at a reasonable price) | 3 | | |  | |  | | | 1 | |  | 1 | |  | |  |  |  | |  | | 1 | Keller 2007 |
| Easy access to the program by bringing the program to participants | 1 | | | 1 | |  | | |  | |  |  | |  | |  | 1 |  | | 1 | |  | Wierenga 2013 |
|  | 2 | | | 1 | |  | | |  | |  |  | |  | | 1 |  | 1 | |  | |  | Koplan 2005 |
| Equal access to physical activity and sports opportunities, especially for people living in rural or deprived areas | 3 | | |  | | 1 | | |  | |  | 1 | |  | |  |  |  | |  | | 1 | Christiansen 2012 |
| *Evaluating and solving time-related issues in implementation* | | | | | | | | | | | | | | | | | | | | | | | |
| Lack of time in the community involved in implementation | 1 | | | 1 | |  | | |  | |  |  | |  | | 1 |  |  | | 1 | |  | Doak 2006 |
|  | 2 | | | 1 | |  | | |  | |  | 1 | |  | |  |  | 1 | |  | |  | WHO Europe 2007a |
|  | 3 | | |  | | 1 | | | 1 | |  | 1 | |  | | 1 |  |  | | 1 | | 1 | Caraher 2007; Naylor 2008 |
| Sufficient time available to the implementers (in context to their workload) to implement intervention | 1 | | | 2 | | 1 | | |  | |  |  | |  | | 1 | 2 |  | | 3 | |  | Wierenga 2013; Benjaminmin 2014; Doak 2006 |
| Time constraints of participants | 1 | | | 1 | |  | | |  | |  |  | |  | |  | 1 |  | | 1 | |  | Wierenga 2013 |
|  | 2 | | | 1 | |  | | |  | |  |  | |  | | 1 |  | 1 | |  | |  | Koplan 2005 |
|  | 3 | | | 1 | |  | | |  | |  |  | |  | | 1 |  | 1 | |  | |  | Larson 2012 |
| Time for implementation: assessment of time needed for implementation conducted and adequate time secured | 1 | | | 1 | |  | | |  | |  |  | |  | |  | 1 |  | | 1 | |  | Wierenga 2013 |
|  | 2 | | | 2 | |  | | |  | |  | 1 | |  | | 1 |  | 1 | |  | | 1 | CDC 2011; WHO Europe 2007a |
|  | 3 | | |  | |  | | | 1 | |  | 1 | |  | |  |  |  | |  | | 1 | Caraher 2007 |
| Time investment without financial compensation | 1 | | |  | | 1 | | |  | |  |  | |  | |  | 1 |  | | 1 | |  | Goodwin 2011 |
| Timing of intervention activities: intervention activities coincide with other activities (e.g., scheduled breaks) | 1 | | | 1 | |  | | |  | |  |  | |  | |  | 1 |  | | 1 | |  | Wierenga 2013 |
| A lack of time for evaluation | 2 | | | 1 | |  | | |  | |  |  | |  | | 1 |  | 1 | |  | |  | Koplan 2005 |
| Limited time in curriculum to add new program in respective settings (e.g., schools) | 1 | | | 1 | |  | | |  | |  |  | |  | | 1 |  |  | | 1 | |  | Doak 2006 |
|  | 3 | | |  | | 2 | | | 1 | |  | 2 | |  | | 1 |  | 1 | | 1 | | 1 | Shill 2012a; Naylor 2008; Kahn 2002 |
| Time: Project implementation took more time than expected due to high workload of administration and planning | 1 | | | 1 | |  | | |  | |  |  | |  | |  | 1 |  | | 1 | |  | Wierenga 2013 |
| Time needed for implementation: assessment conducted | 3 | | |  | |  | | | 1 | |  | 1 | |  | |  |  |  | |  | | 1 | Caraher 2007 |
| Adequate time for implementation of the intervention among all participants secured | 2 | | | 1 | |  | | |  | |  | 1 | |  | |  |  | 1 | |  | |  | WHO Europe 2007a |
| *Fidelity* | | | | | | | | | | | | | | | | | | | | | | | |
| Fidelity of the program (in reference to the content and the dose of the program) | 1 | | | 3 | | 2 | | |  | |  | 1 | |  | | 1 | 3 |  | | 5 | |  | Goodwin 2011; McMahon 2012; Wierenga 2013; Goode 2012; Langford 2014 |
|  | 2 | | | 1 | |  | | | 1 | |  | 1 | |  | | 1 |  |  | | 1 | | 1 | Stallings 2010; Kumanyika 2010 |
|  | 3 | | | 1 | |  | | |  | |  | 1 | |  | |  |  | 1 | |  | |  | Glasgow 2007 |
| Evaluate intervention delivery via an independent observer | 1 | | |  | | 1 | | |  | |  |  | |  | |  | 1 |  | | 1 | |  | McMahon 2012 |
|  | 3 | | |  | | 2 | | |  | |  | 1 | | 1 | |  |  |  | | 1 | | 1 | Antikainen 2011; Craig 2012 |
| The use of protocol/following the protocol | 1 | | | 1 | |  | | |  | |  |  | |  | |  | 1 |  | | 1 | |  | Ghisi 2014 |
|  | 3 | | | 1 | | 1 | | |  | |  | 1 | |  | |  | 1 |  | | 2 | |  | Cambon 2012; White 2009 |
| Degree to which intervention is delivered as intended (compared to the protocol) | 1 | | | 1 | | 2 | | |  | |  | 2 | |  | |  | 1 |  | | 3 | |  | Blackman 2013; Matthews 2014; Galaviz 2014 |
|  | 3 | | |  | | 1 | | |  | |  |  | |  | |  | 1 |  | | 1 | |  | White 2009 |
| Assessment of fidelity of delivery | 1 | | | 3 | | 1 | | |  | |  | 2 | |  | |  | 2 |  | | 4 | |  | Goode 2012; Galaviz 2014; Laws 2012; Matthews 2014 |
| Various methods used for assessing intervention fıdelity | 1 | | | 1 | | 1 | | |  | |  | 2 | |  | |  |  |  | | 2 | |  | Goode 2012; Galaviz 2014 |
| Dose delivered versus dose received analysis | 1 | | | 1 | |  | | |  | |  |  | |  | |  | 1 |  | | 1 | |  | Wierenga 2013 |
| Regular meetings with staff to evaluate the fidelity of implementation | 1 | | | 1 | |  | | |  | |  |  | |  | |  | 1 |  | | 1 | |  | Matthews 2014 |
| Fully developed intervention protocol including core components that are essential to deliver | 1 | | |  | | 1 | | |  | |  | 1 | |  | |  |  |  | | 1 | |  | Huijg 2014 |
| Assessment of the quality of implementation (for example, lesson quality) | 1 | | | 1 | |  | | |  | |  |  | |  | | 1 |  |  | | 1 | |  | Langford 2014 |
|  | 3 | | | 1 | |  | | |  | |  | 1 | |  | |  |  | 1 | |  | |  | King 2011 |
| *Use of implementation theory/framework* | | | | | | | | | | | | | | | | | | | | | | | |
| The use of implementation theory for implementation practice | 1 | | | 2 | | 2 | | |  | |  | 2 | | 1 | | 1 |  | 1 | | 3 | |  | Ickes 2013; Rabin 2010; Fleury 2009; Baker 2011 |
|  | 3 | | | 1 | |  | | |  | |  | 1 | |  | |  |  |  | | 1 | |  | Cambon 2012 |
| Use of RE-AIM framework for identification, appraisal, and synthesis of material | 1 | | | 4 | | 4 | | |  | |  | 2 | | 1 | | 1 | 4 | 1 | | 7 | |  | Mcmahon 2012; Akers 2010; Blackman 2013; Eakin 2002; Vuillemin 2011; Galaviz 2014; Brennan 2014; Kohl 2013 |
|  | 3 | | | 2 | | 3 | | |  | |  | 2 | | 1 | |  | 2 | 1 | | 4 | |  | Eakin 2005;  Dzewaltowski 2004; White 2009; Antikainen 2011; Besculides 2008 |
| *Cultural context in implementation* | | | | | | | | | | | | | | | | | | | | | | | |
| Culture-sensitive implementation, addressing the needs of diverse population in their community context (social, cultural, economic, and political) | 1 | | | 3 | | 2 | | | 2 | |  | 2 | | 2 | | 1 | 2 | 3 | | 3 | | 1 | Mcmahon 2012; Waters 2011; Nierkens 2013; Sahay 2006; Carroll 2011; Fleury 2009; Teufel-Shone 2009; Niebylski 2014 |
|  | 2 | | | 4 | | 3 | | | 1 | |  | 3 | |  | | 4 | 1 | 2 | |  | | 6 | WHO Europe 2007a; NICE 2012; Stallings 2010; Sims 2006; WHO 2008; CDC 2011; WHO 2010, NICE 2009 |
|  | 3 | | | 3 | | 3 | | | 3 | |  | 4 | |  | | 4 | 1 | 2 | | 4 | | 3 | Larson 2012; Naylor 2013; Ribeiro 2010; Sorensen 2004; Kahn 2002; Cambon 2012; Keller 2007; Ramanathan 2008; Thow 2011 |
| Deep-structure adaptations (cultural adaptations to participants, consultations with community advisor on cultural adaptations, consultation with participants) | 1 | | | 1 | |  | | |  | |  |  | | 1 | |  |  |  | | 1 | |  | Nierkens 2013 |
|  | 2 | | |  | | 1 | | |  | |  | 1 | |  | |  |  |  | |  | | 1 | WHO 2010 |
| Characteristics of the socio-political context: compatibility of program with societal changes (attention for health in society) | 1 | | | 1 | |  | | |  | |  |  | |  | |  | 1 |  | | 1 | |  | Wierenga 2013 |
| Training for staff in processes of cultural adaptation | 1 | | | 1 | |  | | |  | |  |  | | 1 | |  |  |  | | 1 | |  | Nierkens 2013 |
| Culturally sensitive approaches within a common framework (e.g., secured by layered flexibility) | 3 | | |  | | 1 | | |  | |  | 1 | |  | |  |  |  | |  | | 1 | Craig 2012 |
| *Cost and funding of implementation* | | | | | | | | | | | | | | | | | | | | | | | |
| Costs of implementation analyzed (e.g., analysis of costs to deliver per person) | 1 | | | 4 | | 3 | | | 1 | |  | 2 | |  | | 3 | 3 | 2 | | 6 | |  | Mcmahon 2012; Akers 2010; Blackman 2013; Galaviz 2014; Wang 2012; Matthews 2014; Waters 2011; Langford 2014 |
|  | 3 | | |  | |  | | | 1 | |  | 1 | |  | |  |  |  | |  | | 1 | Caraher 2007 |
| Funding/resources for implementation secured and provided | 1 | | | 3 | | 2 | | |  | |  | 1 | |  | | 4 |  | 3 | | 1 | | 1 | Huijg 2014; Robertson-Wilson 2012; Waters 2011; Hearn 2008, Flynn 2006 |
|  | 2 | | | 3 | | 2 | | | 1 | |  | 4 | |  | | 2 |  | 1 | |  | | 5 | WHO Europe 2007a; WHO Europe 2007b; WHO 2007; WHO 2008; WHO 2013 CDC 2011 |
|  | 3 | | | 1 | | 2 | | |  | |  | 3 | |  | |  |  |  | | 1 | | 2 | Bellew 2008; Christiansen 2012; Cambon 2012 |
| Lack of/limited funding for implementation | 1 | | |  | | 2 | | |  | |  |  | |  | |  | 2 |  | | 2 | |  | Benjamin 2014; Goodwin 2011 |
|  | 2 | | | 1 | |  | | |  | |  |  | |  | |  | 1 |  | | 1 | |  | EC 2006 |
|  | 3 | | | 1 | | 2 | | | 2 | |  | 4 | |  | | 1 |  | 1 | |  | | 4 | Fransen 2012; Carlsson 2008; Christiansen 2012; Craig 2012; Caraher 2007 |
| Uncertainly about funding in changing legal (policy) context, recession | 3 | | |  | | 1 | | | 1 | |  | 2 | |  | |  |  |  | |  | | 2 | Caraher 2007; Craig 2012 |
| Costs/funding for sustainable maintenance | 1 | | |  | | 1 | | |  | |  |  | | 1 | |  |  | 1 | |  | |  | Teufel-Shone 2009 |
|  | 3 | | |  | | 2 | | |  | |  | 2 | |  | |  |  |  | |  | | 2 | Shill 2012b; Bellew 2008 |
| Cost targets: low (feasible) costs of implementation, cheap resources, and affordable across settings | 1 | | | 3 | |  | | |  | |  |  | | 1 | | 1 | 1 | 1 | | 2 | |  | Brennan 2014; Van De Vijver 2012; Cardona-Morrell 2010 |
|  | 2 | | |  | |  | | | 1 | |  |  | |  | | 1 |  |  | |  | | 1 | Stallings 2010 |
|  | 3 | | | 1 | | 1 | | |  | |  |  | |  | | 1 | 1 | 1 | |  | | 1 | Tremblay 2012; Besculides 2008 |
| Payment for delivery agents | 1 | | |  | | 1 | | |  | |  | 1 | |  | |  |  | 1 | |  | |  | Hoehner 2013 |
| Compensation for the participation of professionals and beneficiaries | 3 | | | 1 | |  | | |  | |  | 1 | |  | |  |  |  | | 1 | |  | Cambon 2012 |
| Funding needed for staff hours required | 1 | | | 1 | |  | | |  | |  |  | |  | | 1 |  | 1 | |  | |  | Waters 2011 |
| Securing funds for personnel involved in implementation | 1 | | | 1 | | 1 | | |  | |  | 1 | |  | | 1 |  | 2 | |  | |  | Hoehner 2013; Waters 2011 |
|  | 3 | | | 1 | |  | | |  | |  | 1 | |  | |  |  |  | | 1 | |  | Cambon 2012 |
| Costs of maintenance measured | 1 | | |  | | 1 | | |  | |  | 1 | |  | |  |  |  | | 1 | |  | Galaviz 2014 |
| Plans for budget sustainability: plans/facilitation for funding after program finished | 1 | | | 1 | |  | | |  | |  |  | | 1 | |  |  |  | | 1 | |  | Fleury 2009 |
| Bids for national level/governmental funding to secure long-term funding | 2 | | | 1 | |  | | |  | |  |  | |  | | 1 |  | 1 | |  | |  | Koplan 2005 |
|  | 3 | | |  | |  | | | 1 | |  | 1 | |  | |  |  |  | |  | | 1 | Caraher 2007 |
| Securing funds for long-term maintenance (e.g., through national government funds) | 1 | | | 1 | | 1 | | |  | |  | 1 | | 1 | |  |  |  | | 2 | |  | Fleury 2009; Galaviz 2014 |
|  | 2 | | | 1 | |  | | |  | |  |  | |  | | 1 |  | 1 | |  | |  | Koplan 2005 |
|  | 3 | | |  | |  | | | 1 | |  | 1 | |  | |  |  |  | |  | | 1 | Caraher 2007 |
| Funds for monitoring/evaluation secured | 2 | | | 2 | |  | | |  | |  | 1 | |  | | 1 |  | 1 | |  | | 1 | Koplan 2005; NICE 2012 |
| Affordable across clinical care settings | 1 | | | 1 | |  | | |  | |  |  | |  | |  | 1 |  | | 1 | |  | Cardona-Morrell 2010 |
| Costs of environmental changes (remodelling/refitting the environment) | 1 | | |  | | 1 | | |  | |  | 1 | |  | |  |  | 1 | |  | |  | Heath 2006 |
|  | 3 | | |  | | 1 | | |  | |  | 1 | |  | |  |  |  | |  | | 1 | Shill 2012b |
| Transparency of funding for implementation (explicit sources) | 3 | | | 1 | |  | | |  | |  | 1 | |  | |  |  | 1 | |  | |  | King 2011 |
| Difficulty to meet different funding stream demands | 3 | | |  | |  | | | 1 | |  | 1 | |  | |  |  |  | |  | | 1 | Caraher 2007 |
| *Other resources needed for delivery* | | | | | | | | | | | | | | | | | | | | | | | |
| Lack of resources for implementation in organizations involved in delivery | 1 | | | 2 | | 1 | | |  | |  |  | |  | | 2 | 1 | 1 | | 1 | | 1 | Wierenga 2013; Waters 2011;  Robertson-Wilson 2012 |
|  | 3 | | |  | | 1 | | | 1 | |  |  | |  | | 2 |  |  | | 1 | | 1 | Carlsson 2008; Naylor 2008 |
| Resources required to secure the implementation across its duration | 1 | | | 1 | |  | | |  | |  |  | |  | | 1 |  | 1 | |  | |  | Hearn 2008 |
|  | 2 | | |  | |  | | | 1 | |  |  | |  | | 1 |  |  | |  | | 1 | Stallings 2010 |
|  | 3 | | | 1 | |  | | |  | |  | 1 | |  | |  |  |  | | 1 | |  | Cambon 2012 |
| Lack of resources for implementation (from sources other than involved organizations) | 1 | | |  | | 2 | | |  | |  | 2 | |  | |  |  |  | | 2 | |  | Huijg 2014; Galaviz 2014 |
|  | 2 | | | 1 | |  | | |  | |  |  | |  | | 1 |  |  | |  | | 1 | CDC 2011 |
|  | 3 | | | 1 | | 1 | | |  | |  | 2 | |  | |  |  | 1 | |  | | 1 | Fransen 2012; Craig 2012 |
| Availability of resources in organization for routine application of the intervention | 1 | | | 1 | |  | | |  | |  |  | |  | |  | 1 |  | | 1 | |  | Matthews 2014 |
|  | 3 | | | 1 | |  | | | 1 | |  | 2 | |  | |  |  | 1 | | 1 | |  | Cambon 2012; Stockley 2001 |
| Adequate resources secured for implementation | 1 | | | 1 | |  | | |  | |  |  | |  | | 1 |  | 1 | |  | |  | Hearn 2008, |
|  | 3 | | |  | | 2 | | |  | |  | 2 | |  | |  |  | 1 | |  | | 1 | Bellew 2008; Kahn 2002 |
| Technical problems (e.g., equipment breaks down) | 1 | | | 1 | |  | | |  | |  |  | |  | |  | 1 |  | | 1 | |  | Wierenga 2013 |
| *Delivery characteristics* | | | | | | | | | | | | | | | | | | | | | | | |
| Documentation and reporting of the processes by which interventions are delivered | 1 | | | 1 | |  | | |  | |  | 1 | |  | |  |  |  | | 1 | |  | Goode 2012 |
|  | 3 | | |  | | 2 | | |  | |  | 1 | | 1 | |  |  |  | | 1 | | 1 | Antikainen 2011;  Christiansen 2012 |
| Extent to which protocol was delivered as intended/protocol adherence | 1 | | | 3 | | 2 | | |  | |  | 1 | |  | | 2 | 2 | 1 | | 4 | |  | Akers 2010; Blackman 2013; Waters 2011; Beets 2009; Kohl 2013 |
|  | 3 | | | 1 | | 1 | | |  | |  | 2 | |  | |  |  |  | | 2 | |  | Eakin 2005; Dzewaltowski 2004 |
| Consistency of delivery and evaluation/monitoring of consistency | 1 | | | 2 | | 3 | | |  | |  | 2 | |  | | 1 | 2 | 1 | | 4 | |  | Klesges 2008; Vuillemin 2011; Galaviz 2014; Laws 2012; Heath 2012 |
|  | 3 | | | 2 | |  | | | 1 | |  | 3 | |  | |  |  | 1 | | 1 | | 1 | King 2011; Dzewaltowski 2004; Keller 2007 |
| Identifying the essential amount of time/number of sessions required to deliver the program | 1 | | | 3 | | 1 | | |  | |  |  | |  | | 1 | 3 | 1 | | 3 | |  | Klesges 2008; Matthews 2014; Vuillemin 2011; Laws 2012 |
|  | 3 | | |  | | 2 | | |  | |  | 2 | |  | |  |  |  | | 1 | | 1 | Eakin 2005; Christiansen 2012 |
| Setting goals/targets for implementation | 2 | | | 1 | | 2 | | |  | |  | 1 | |  | | 1 | 1 |  | |  | | 3 | Sims 2006; WHO Europe 2007b; WHO 2008 |
| Mass media involved in delivery and dissemination | 2 | | | 1 | | 1 | | |  | |  | 1 | |  | |  | 1 |  | | 1 | | 1 | WHO 2007; EC 2006 |
|  | 3 | | |  | |  | | | 3 | |  | 3 | |  | |  |  |  | |  | | 3 | Keller 2007; Perez-Ferrer 2010; Shill 2012a |
| Use mass media to delivery (using a website and provided access for individuals to pedometers and logbooks) | 1 | | |  | | 1 | | |  | |  | 1 | |  | |  |  | 1 | |  | |  | Baker 2011 |
| Local delivery/implementation plan | 3 | | |  | |  | | | 1 | |  | 1 | |  | |  |  |  | |  | | 1 | Caraher 2007 |
| Specific action plans for implementation steps | 3 | | |  | | 1 | | |  | |  | 1 | |  | |  |  |  | |  | | 1 | Christiansen 2012 |
| Planning systems for implementation | 3 | | |  | | 1 | | |  | |  | 1 | |  | |  |  |  | |  | | 1 | Shill 2012b |
| Plans for delivery (plans for coordination, communication, specific steps, specific settings) | 3 | | |  | | 2 | | | 1 | |  | 3 | |  | |  |  |  | |  | | 3 | Shill 2012b Christiansen 2012; Caraher 2007 |
| Involving any available staff into the program delivery | 1 | | | 1 | | 2 | | |  | |  |  | | 2 | | 1 |  | 1 | | 2 | |  | Ickes 2013; Verstraeten 2012; Teufel-Shone 2009 |
|  | 3 | | |  | | 1 | | |  | |  |  | |  | | 1 |  |  | | 1 | |  | Naylor 2008 |
| Delivery through various professional groups | 1 | | | 1 | |  | | |  | |  |  | |  | | 1 |  | 1 | |  | |  | Klesges 2008 |
|  | 3 | | |  | | 2 | | |  | |  | 2 | |  | |  |  |  | | 1 | | 1 | Eakin 2005; Craig 2012 |
| Clear identification of roles and responsibilities in implementation processes | 2 | | | 2 | |  | | |  | |  |  | |  | | 1 | 1 |  | | 1 | | 1 | CDC 2011; EC 2006 |
|  | 3 | | |  | | 2 | | |  | |  | 2 | |  | |  |  |  | |  | | 2 | Craig 2012; Bellew 2008 |
| Lay health advisors involved in delivery | 1 | | | 1 | |  | | |  | |  |  | | 1 | |  |  |  | | 1 | |  | Fleury 2009 |
| Appointment of a high profile advising agency to implement policy | 3 | | | 1 | |  | | |  | |  |  | |  | | 1 |  |  | |  | | 1 | Freudenberg 2010 |
| Users involved in implementation | 3 | | |  | | 1 | | | 2 | |  | 1 | |  | | 1 | 1 | 1 | | 2 | |  | Naylor 2008; Sorensen 2004; Stockley 2001 |
| Health professionals involved in delivery and supporting implementation | 3 | | |  | | 1 | | |  | |  |  | |  | | 1 |  |  | | 1 | |  | Naylor 2008 |
| Delegation of responsibilities in implementation process | 3 | | |  | | 1 | | |  | |  | 1 | |  | |  |  |  | |  | | 1 | Christiansen 2012 |
| Delivery through various professional groups, lay health advisors, and users | 1 | | | 1 | | 1 | | |  | |  |  | | 1 | | 1 |  |  | | 2 | |  | Ickes 2013; Fleury 2009 |
|  | 3 | | | 1 | | 3 | | | 1 | |  | 2 | |  | | 2 | 1 |  | | 2 | | 3 | Craig 2012; Freudenberg 2010; Christiansen 2012; Naylor 2008; Sorensen 2004 |
| Pilots: testing new and existing materials before delivering to the target population | 1 | | | 1 | |  | | |  | |  |  | | 1 | |  |  |  | | 1 | |  | Verstraeten 2012 |
|  | 3 | | | 3 | |  | | | 1 | |  | 1 | | 1 | | 1 | 1 | 1 | | 2 | | 1 | Gaziano 2007; Summerbell 2012; King 2011; Sorensen 2004 |
| Quality monitoring | 1 | | | 1 | |  | | |  | |  |  | |  | | 1 |  |  | | 1 | |  | Langford 2014 |
|  | 3 | | | 1 | |  | | |  | |  | 1 | |  | |  |  | 1 | |  | |  | King 2011 |
| Implementation manual | 3 | | |  | |  | | | 1 | |  | 1 | |  | |  |  |  | |  | | 1 | Keller 2007 |
| Evaluation of continuity of delivery | 1 | | |  | | 1 | | |  | |  |  | |  | |  | 1 |  | | 1 | |  | McMahon 2012 |
|  | 3 | | | 1 | |  | | |  | |  | 1 | |  | |  |  |  | | 1 | |  | Cambon 2012 |
| Degree to which participants received intervention components | 1 | | |  | | 2 | | |  | |  | 2 | |  | |  |  |  | | 2 | |  | Blackman 2013; Galaviz 2014 |
| Variability and heterogeneity in implementation across regions | 3 | | | 1 | |  | | |  | |  |  | |  | | 1 |  |  | |  | | 1 | Ramanathan 2008 |
| Private sector used for dissemination of policy | 2 | | |  | | 1 | | |  | |  | 1 | |  | |  |  |  | |  | | 1 | WHO 2007 |
|  | 3 | | |  | |  | | | 1 | |  | 1 | |  | |  |  |  | |  | | 1 | Keller 2007 |
| *Settings’ characteristics affecting delivery and implementation* | | | | | | | | | | | | | | | | | | | | | | | |
| Organizational practices supporting implementation, management participation in implementation | 1 | | | 1 | |  | | |  | |  |  | |  | |  | 1 |  | | 1 | |  | Wierenga 2013 |
|  | 2 | | | 2 | | 1 | | |  | |  |  | |  | | 2 | 1 | 2 | |  | | 1 | Birch 2011; NICE 2008; Koplan 2005 |
|  | 3 | | |  | |  | | | 1 | |  |  | |  | |  | 1 |  | | 1 | |  | Sorensen 2004 |
| Organizational culture (e.g., goal setting and tracks progress towards achieving goals) facilitating implementation | 1 | | | 1 | |  | | | 1 | |  |  | |  | | 1 | 1 | 1 | | 1 | |  | Wierenga 2013; Wang2012 |
| Aims and existing polices within the organization are accounted for (how does the program fit into organizational aims and existing policies?) | 1 | | | 1 | |  | | | 1 | |  |  | |  | | 1 | 1 | 1 | | 1 | |  | Wierenga 2013; Wang 2012 |
|  | 2 | | | 2 | |  | | |  | |  | 1 | |  | | 1 |  | 1 | |  | | 1 | NICE 2008; Koplan 2005 |
| Management support for implementation | 1 | | | 1 | |  | | |  | |  |  | |  | |  | 1 |  | | 1 | |  | Wierenga 2013 |
|  | 3 | | |  | |  | | | 1 | |  |  | |  | |  | 1 |  | | 1 | |  | Sorensen 2004 |
| Organizational awareness and image (orientation towards well-being of students/employees) | 1 | | | 1 | |  | | |  | |  |  | |  | |  | 1 |  | | 1 | |  | Wierenga 2013 |
|  | 3 | | |  | |  | | | 1 | |  |  | |  | |  | 1 |  | | 1 | |  | Sorensen 2004 |
| Identifying optimal intervention environment (comfort) | 3 | | | 1 | |  | | |  | |  | 1 | |  | |  |  |  | | 1 | |  | Cambon 2012 |
| Characteristics of the interventions setting - the uniqueness of setting | 3 | | | 1 | |  | | |  | |  | 1 | |  | |  |  |  | | 1 | |  | Cambon 2012 |
| Changes/turnover in administration/staff in organizations | 2 | | | 1 | |  | | |  | |  |  | |  | | 1 |  |  | |  | | 1 | CDC 2011 |
| Existing institutional frameworks for implementation | 3 | | |  | |  | | | 1 | |  |  | |  | | 1 |  |  | |  | | 1 | Bundy 2012 |
| *Adjustments and customizations in implementation* | | | | | | | | | | | | | | | | | | | | | | | |
| Deep-structure adaptations (deep cultural and ethnic adaptations to participants, consultations with community advisors on cultural adaptations, consultation with participants) | 1 | | | 2 | |  | | |  | |  |  | | 1 | |  | 1 |  | | 2 | |  | Nierkens 2013; Matthews 2014 |
|  | 2 | | | 1 | | 1 | | | 1 | |  | 2 | |  | | 1 |  |  | | 1 | | 2 | WHO 2010; Stallings 2010; Kumanyika 2010 |
|  | 3 | | | 1 | |  | | |  | |  |  | |  | | 1 |  |  | | 1 | |  | Summerbell 2012 |
| Customization of the program (to target population and local conditions) | 1 | | | 3 | | 1 | | |  | |  |  | |  | | 2 | 2 | 2 | | 2 | |  | Klesges 2012; Klesges 2008; Vuillemin 2011; Kohl 2013 |
|  | 2 | | | 1 | |  | | |  | |  | 1 | |  | |  |  |  | | 1 | |  | Kumanyika 2010 |
|  | 3 | | | 4 | | 2 | | |  | |  | 4 | | 1 | |  | 1 | 4 | | 2 | |  | Cambon 2012; Glasgow 2007; Antkainen 2011; Besculides 2008; Kahn 2002; King 2011 |
| Potential adaptations to enhance the fıt within community contexts | 1 | | | 4 | | 1 | | |  | |  | 1 | |  | | 2 | 2 | 2 | | 3 | |  | Goode 2012; Klesges 2012; Klesges 2008; Vuillemin 2011; Kohl 2013 |
|  | 2 | | | 1 | |  | | | 1 | |  | 1 | |  | | 1 |  |  | | 1 | | 1 | Stallings 2010; Kumanyika 2010 |
|  | 3 | | | 1 | | 1 | | |  | |  | 2 | |  | |  |  | 2 | |  | |  | Kahn 2002; King 2011 |
| Adjustments to participants needs | 1 | | | 1 | | 1 | | |  | |  | 1 | | 1 | |  |  |  | | 2 | |  | Huijg 2014; Verstraeten 2012 |
|  | 2 | | |  | | 1 | | |  | |  |  | |  | | 1 |  | 1 | |  | |  | NICE 2009 |
| Assessment of adaptations of the intervention/policy made during delivery | 1 | | | 3 | |  | | |  | |  | 1 | |  | | 2 |  | 1 | | 2 | |  | Rabin 2010; Brennan 2014; Langford 2014 |
|  | 3 | | | 1 | |  | | |  | |  | 1 | |  | |  |  | 1 | |  | |  | Glasgow 2007 |
| Adoption to settings | 1 | | | 4 | |  | | | 1 | |  |  | | 1 | | 2 | 2 | 2 | | 3 | |  | Klesges 2012; Verstraeten 2012; Wierenga 2013; Kohl 2013; Wang 2012 |
| Customization of the program: adjustments to staff expertise | 1 | | | 1 | | 2 | | |  | |  | 1 | |  | | 1 | 1 | 1 | | 2 | |  | Klesges 2012; Huijg 2014; Goodwin 2011 |
| Adjustments to meet the needs of organizations and practitioners | 1 | | |  | | 1 | | |  | |  |  | |  | |  | 1 |  | | 1 | |  | Goodwin 2011 |
| Adjustments to primary care/general practice management | 1 | | |  | | 1 | | |  | |  |  | |  | |  | 1 |  | | 1 | |  | Goodwin 2011 |
| Degree of incorporation of program communication and into already established communication channels or existing worksite events/meetings | 1 | | | 2 | |  | | |  | |  |  | |  | |  | 2 |  | | 2 | |  | Wierenga 2013; Kohl 2013 |
| The scale of modifications made (original trial to other real life practice) | 1 | | | 1 | |  | | |  | |  |  | |  | |  | 1 |  | | 1 | |  | Cardona-Morrell 2010 |
| Which components are adjusted/modified over time? | 1 | | |  | | 1 | | |  | |  |  | |  | |  | 1 |  | | 1 | |  | Vuillemin 2011 |
| Revisiting programs as they evolve | 3 | | |  | |  | | | 1 | |  |  | |  | | 1 |  |  | |  | | 1 | Bundy 2012 |
| Planning for adaptations | 3 | | |  | | 1 | | |  | |  | 1 | |  | |  |  | 1 | |  | |  | Kahn 2002 |
| Monitoring and feedback on adjustments/adaptations of the project | 3 | | |  | |  | | | 1 | |  | 1 | |  | |  |  | 1 | |  | |  | Stockley 2001 |
| Developing policies allowing for flexibility | 1 | | |  | | 1 | | |  | |  |  | |  | | 1 |  |  | |  | | 1 | Robertson-Wilson 2012 |
| Layered flexibility | 3 | | |  | | 1 | | |  | |  | 1 | |  | |  |  |  | |  | | 1 | Craig 2012 |
| Interventions allowing for greater flexibility are more likely to have broader reach | 3 | | |  | | 1 | | |  | |  |  | |  | |  | 1 |  | | 1 | |  | White 2009 |
| Consult community and reorient the project (e.g., if community not involved) | 3 | | |  | |  | | | 1 | |  | 1 | |  | |  |  |  | |  | | 1 | Caraher 2007 |
| Intervention should be integrated into the school curriculum | 1 | | | 2 | |  | | |  | |  |  | | 1 | | 1 |  |  | | 2 | |  | Verstraeten 2012; Langford 2014 |
|  | 3 | | | 1 | |  | | |  | |  |  | |  | | 1 |  |  | | 1 | |  | Summerbell 2012 |
| Flexibility in program implementation | 3 | | |  | |  | | | 1 | |  |  | |  | |  | 1 |  | | 1 | |  | Sorensen 2004 |
| *Planning and monitoring of implementation processes* | | | | | | | | | | | | | | | | | | | | | | | |
| Plans for implementation | 1 | | | 2 | | 1 | | | 1 | |  | 3 | |  | | 1 |  | 2 | | 2 | |  | Rabin 2010; Waters 2011; Sahay 2006; Heath 2012 |
|  | 3 | | | 2 | |  | | |  | |  |  | |  | | 1 | 1 | 1 | | 1 | |  | Besculides 2008; Taylor 2013 |
| Plans for monitoring and plans for evaluation (how to increase data availability and of high quality?) | 2 | | | 2 | |  | | |  | |  |  | |  | | 2 |  |  | |  | | 2 | WHO 2008; CDC 2011; |
|  | 3 | | | 1 | | 1 | | | 1 | |  | 2 | |  | | 1 |  |  | | 1 | | 2 | Taylor 2013; Capacci 2012; Christiansen 2012 |
| Process monitoring and evaluation | 1 | | | 3 | |  | | |  | |  |  | | 2 | | 1 |  | 1 | | 2 | |  | Waters 2011; van De Vijver 2012; Verstraeten 2012 |
|  | 2 | | | 2 | |  | | | 1 | |  | 2 | |  | |  | 1 | 1 | | 1 | | 1 | WHO Europe 2007a, WHO 2013; EC 2006 |
|  | 3 | | | 1 | | 1 | | | 1 | |  | 1 | |  | | 1 | 1 |  | | 1 | | 2 | Keller 2007; Ramanathan 2008; White 2009 |
| Monitoring and assessment of adherence to implementation protocol/protocol fidelity | 1 | | | 2 | |  | | |  | |  |  | |  | |  | 2 |  | | 2 | |  | Ghisi 2014; Kohl 2013 |
|  | 2 | | | 1 | | 1 | | |  | |  | 1 | |  | | 1 |  | 2 | |  | |  | WHO Europe 2007a; NICE 2009 |
|  | 3 | | | 1 | |  | | |  | |  | 1 | |  | |  |  | 1 | |  | |  | Glasgow 2007 |
| Monitoring of progress & achievements, documenting results | 2 | | |  | |  | | | 1 | |  | 1 | |  | |  |  |  | |  | | 1 | WHO 2013 |
| Reaching a target of ‘successful implementation’ is measured (specific criteria, targets, timeframe) | 3 | | |  | | 1 | | |  | |  | 1 | |  | |  |  |  | |  | | 1 | Bellew 2008 |
| Independent monitoring system for evaluation of processes | 3 | | |  | | 1 | | |  | |  | 1 | |  | |  |  |  | |  | | 1 | Craig 2012 |
| Degree of implementation across settings evaluated | 1 | | | 1 | |  | | |  | |  |  | |  | | 1 |  |  | | 1 | |  | Langford 2014 |
| Monitoring of food quality (in locations where food policy implemented) | 2 | | |  | |  | | | 1 | |  |  | |  | | 1 |  |  | |  | | 1 | Stallings 2010 |
| *Participants’ characteristics affecting implementation* | | | | | | | | | | | | | | | | | | | | | | | |
| Needs of participants exposed to a program (expectations and preferences referring to the program) | 1 | | | 2 | |  | | |  | |  |  | |  | | 1 | 1 |  | | 2 | |  | Wierenga 2013; Doak 2006 |
|  | 3 | | | 1 | |  | | |  | |  |  | |  | | 1 |  |  | | 1 | |  | Taylor 2013 |
| Attitudes towards program and knowledge of participants | 1 | | | 1 | | 1 | | |  | |  |  | |  | | 2 |  | 1 | | 1 | |  | Hearn 2008; Beets 2009 |
| Current workload, work demands, social support at work (for the program) among participants | 1 | | | 1 | |  | | |  | |  |  | |  | |  | 1 |  | | 1 | |  | Wierenga 2013 |
| Participants’ self-efficacy and social support available | 1 | | | 1 | |  | | |  | |  |  | |  | |  | 1 |  | | 1 | |  | Wierenga 2013 |
|  | 3 | | | 1 | |  | | |  | |  |  | |  | |  | 1 | 1 | |  | |  | Besculides 2008 |
| Program perceived as priority by participants | 3 | | | 1 | |  | | |  | |  | 1 | |  | |  |  |  | | 1 | |  | Cambon 2012 |
| Program perceived as creating positive atmosphere among participants | 3 | | | 1 | |  | | |  | |  | 1 | |  | |  |  |  | | 1 | |  | Cambon 2012 |
| Volunteerism and autonomy of participants | 3 | | | 1 | |  | | |  | |  | 1 | |  | |  |  |  | | 1 | |  | Cambon 2012 |
| Cognitive factors and language skills of participants | 3 | | | 1 | |  | | |  | |  | 1 | |  | |  |  |  | | 1 | |  | Cambon 2012 |
| *Implementers’ characteristics affecting implementation* | | | | | | | | | | | | | | | | | | | | | | | |
| Implementers’ expectations regarding the program and perceived control of the program | 1 | | | 1 | | 2 | | |  | |  | 1 | |  | | 1 | 1 |  | | 3 | |  | Wierenga 2013; Huijg 2014; Beets 2009 |
|  | 3 | | |  | | 1 | | | 1 | |  | 1 | |  | | 1 |  |  | |  | | 2 | Tremblay 2012; Caraher 2007 |
| Levels of engagement/involvement and awareness of implementers | 1 | | | 2 | | 1 | | |  | |  |  | |  | | 1 | 2 |  | | 3 | |  | Wierenga 2013; Beets 2009; Laws 2012 |
|  | 2 | | | 1 | |  | | |  | |  |  | |  | | 1 |  | 1 | |  | |  | Koplan 2005; |
|  | 3 | | |  | |  | | | 1 | |  |  | |  | | 1 |  |  | |  | | 1 | Bundy 2012 |
| Support needed (perceived by implementers) | 1 | | | 3 | | 2 | | | 1 | |  | 2 | |  | | 1 | 3 | 2 | | 4 | |  | Wierenga 2013; Goodwin 2011; Huijg 201; Hearn 2008; Sahay 2006; Kohl 2013 |
|  | 3 | | | 1 | |  | | |  | |  | 1 | |  | |  |  | 1 | |  | |  | Gillison 2012 |
| Tendency to comply with the protocol | 1 | | | 1 | |  | | |  | |  |  | |  | |  | 1 |  | | 1 | |  | Wierenga 2013 |
| Number of peer leaders available in respective area where the program is implemented | 1 | | | 1 | |  | | |  | |  |  | |  | |  | 1 |  | | 1 | |  | Wierenga 2013 |
| Enjoyment and satisfaction with implementation | 3 | | | 1 | |  | | |  | |  | 1 | |  | |  |  |  | | 1 | |  | Cambon 2012 |
| Role clarity of the implementer or agencies involved in implementation | 1 | | |  | | 1 | | |  | |  | 1 | |  | |  |  |  | | 1 | |  | Huijg 2014 |
|  | 3 | | |  | | 1 | | |  | |  | 1 | |  | |  |  |  | |  | | 1 | Bellew 2008 |
| Job position (e.g., self-employed, managing own time) | 1 | | | 1 | |  | | |  | |  |  | |  | |  | 1 |  | | 1 | |  | Wierenga 2013 |
| Limited knowledge about setting and organizational processes within (including finances, turnover) | 1 | | | 1 | |  | | |  | |  |  | |  | |  | 1 |  | | 1 | |  | Matthews 2014 |
| ***Domain: Maintenance*** | | | | | | | | | | | | | | | | | | | | | | | |
| *Sustainability* | | | | | | | | | | | | | | | | | | | | | | | |
| Institutionalization of the content of the program and its implementation (e.g., the integration into existing institutional programs) | 1 | | | 2 | | 2 | | |  | |  | 1 | |  | | 1 | 2 | 2 | | 2 | |  | Vuillemin 2011; Klesges 2008; Laws 2012; Hoehner 2013 |
|  | 3 | | | 1 | | 1 | | |  | |  | 1 | | 1 | |  |  | 1 | | 1 | |  | Glasgow 2007; Antikainen 2011 |
| Program maintained over time with institutional support; continuation within the realm of the institution | 1 | | | 1 | | 2 | | |  | |  | 1 | | 1 | | 1 |  | 3 | |  | |  | Brennan 2014; Hoehner 2013; Teufel-Shone 2009 |
| Mutability (intervention/policy is in the community/target group control) | 3 | | | 3 | |  | | |  | |  | 2 | |  | | 1 |  | 3 | |  | |  | Glasgow 2007; McNeil 2007, Khan 2009 |
| Strategies to promote long-term participation (maintenance) included | 1 | | | 1 | | 1 | | |  | |  |  | |  | | 1 | 1 | 2 | |  | |  | Ayliffe 2010; Carroll 2011 |
|  | 3 | | | 2 | |  | | |  | |  | 1 | |  | |  | 1 | 2 | |  | |  | Besculides 2008; Gillison 2012; |
| Building capacity to secure maintenance (training and support in organization, aiming at promotion of maintenance) | 2 | | | 2 | | 2 | | |  | |  | 2 | |  | | 1 | 1 | 1 | |  | | 3 | NICE 2008; NICE 2012; WHO 2007; CDC 2011 |
| Designs /plans for sustainability (in the institution, for specific activities) | 1 | | | 2 | |  | | |  | |  |  | |  | | 1 | 1 | 1 | | 1 | |  | Laws 2012; Brennan 2014 |
|  | 3 | | |  | |  | | | 1 | |  |  | |  | | 1 |  |  | |  | | 1 | Bundy 2012 |
| Multiple reminders to promote/enhance maintenance | 1 | | | 1 | |  | | |  | |  |  | |  | |  | 1 |  | | 1 | |  | Kohl 2013 |
|  | 3 | | | 1 | | 1 | | |  | |  | 1 | |  | |  | 1 | 1 | | 1 | |  | Besculides 2008; Eakin 2005 |
| Newsletters, mails, automated internet reminders to secure maintenance | 3 | | | 1 | | 1 | | |  | |  | 1 | |  | |  | 1 | 1 | | 1 | |  | Besculides 2008; Eakin 2005 |
| Settings related to greater sustainability | 1 | | | 1 | |  | | |  | |  |  | |  | | 1 |  |  | | 1 | |  | Doak 2006 |
|  | 3 | | | 1 | |  | | |  | |  |  | |  | |  | 1 | 1 | |  | |  | Besculides 2008 |
| Sustainability issue - intervention can be continued with minimal financial inputs | 1 | | | 1 | |  | | |  | |  |  | |  | | 1 |  |  | | 1 | |  | Doak 2006 |
| Need to monitor data over decades ( e.g., 20 years) | 3 | | |  | | 1 | | | 1 | |  | 2 | |  | |  |  |  | |  | | 2 | Capacci 2012; Christiansen 2012 |
| Network of organizations involved as means to secure greater sustainability | 1 | | | 1 | |  | | |  | |  |  | |  | |  | 1 |  | | 1 | |  | Matthews 2014 |
| The use of peer recruitment as the strategy securing sustainability | 1 | | | 1 | |  | | |  | |  |  | |  | |  | 1 |  | | 1 | |  | Matthews 2014 |
| Components are institutionalized over time | 1 | | |  | | 1 | | |  | |  |  | |  | |  | 1 |  | | 1 | |  | Vuillemin 2011 |
| Most sustainable components | 1 | | |  | | 1 | | |  | |  |  | |  | |  | 1 |  | | 1 | |  | Vuillemin 2011 |
| Number of sustained components | 1 | | | 1 | |  | | |  | |  |  | |  | | 1 |  | 1 | |  | |  | Brennan 2014 |
| Long-term commitment of organizations as strategy promoting commitment | 2 | | |  | | 1 | | |  | |  |  | |  | |  | 1 | 1 | |  | |  | NICE 2008 |
| Participants’ ‘ownership’ of intervention affects sustainability | 1 | | |  | |  | | | 1 | |  | 1 | |  | |  |  | 1 | |  | |  | Sahay 2006 |
| Governmental funding to promote sustainability - sustained commitment to fund implementation and evaluation | 2 | | | 1 | |  | | |  | |  |  | |  | | 1 |  | 1 | |  | |  | Koplan 2005 |
| The use of programs targeting activities which are a part of regular/normal work, existing resources and compatibility with other programs to secure higher sustainability | 1 | | | 1 | | 1 | | |  | |  |  | |  | |  | 2 |  | | 2 | |  | Goodwin 2011; Wierenga 2013 |
| Sustainability - refers to the need to develop policy procedures and monitoring that are able to deal with the policy problem on a long-term basis. | 3 | | |  | | 1 | | |  | |  | 1 | |  | |  |  |  | |  | | 1 | Rutten 2013 |

Note: The numbers in columns indicate the number of documents addressing respective type of population, type of behavior, policy or intervention and implementation or transferability characteristics addressed in policy/intervention; Document type: 1 = systematic review, 2 = stakeholders’ document, 3 = position review paper; PA – physical activity; for columns ‘Population’, ‘Type of behavior”, ‘Policy/intervention’ and ‘Implementation/transferability’; Vulnerable population - ethnic minorities, various population, indigenous population, underserved population, low and middle income population

**References**

1. Hoelscher DM, Kirk S, Ritchie L, Cunningham-Sabo L, Academy Positions Committee: **Position of the Academy of Nutrition and Dietetics: interventions for the prevention and treatment of pediatric overweight and obesity**. J Acad Nutr Diet. 2013, **113**:1375–1394.
2. Trudnak T, Melton ST, Simpson L, Baldwin J: **The childhood obesity response in Florida: where do we stand?**. Child Obes. 2012, **8**:237–242.
3. Wilding MJ, Seegert L, Rupcic S, Griffin M, Kachnowski S, Parasuraman S: **Falling short: recruiting elderly individuals for a fall study**. Ageing Res Rev. 2013, **12**:552–560.
